# The effects of psychosocial and behavioral interventions on depressive and anxiety symptoms during the COVID-19 pandemic: a systematic review and meta-analysis

**DOI:** 10.1038/s41598-023-45839-0

**Published:** 2023-11-04

**Authors:** Jiali He, Jingxia Lin, Wen Sun, Teris Cheung, Yuan Cao, Eugene Fu, Sunny H. W. Chan, Hector W. H. Tsang

**Affiliations:** 1 Department of Rehabilitation Sciences, The Hong Kong Polytechnic University, Hung Hom, Kowloon, Hong Kong; 2 Mental Health Research Center, The Hong Kong Polytechnic University, Hung Hom, Kowloon, Hong Kong; 3 School of Health and Social Wellbeing, University of the West of England, Bristol, UK; 4 School of Nursing, The Hong Kong Polytechnic University, Hung Hom, Kowloon, Hong Kong

**Keywords:** Psychology, Health care

## Abstract

Psychosocial and behavioral interventions have been shown to significantly reduce depressive and anxiety symptoms in different populations. Recent evidence suggests that the mental health of the general population has deteriorated significantly since the start of Coronavirus Disease 2019 (COVID-19) pandemic. We conducted a systematic review and meta-analysis of studies on the therapeutic effects of psychosocial and behavioral interventions on depression and anxiety during the COVID-19 pandemic. We systematically searched six electronic databases between December 2019 and February 2022 including PubMed, PsycINFO, Scopus, Web of Science, CNKI, and Wanfang Data. We included randomized clinical trials of psychosocial and behavioral interventions in individuals with depressive or anxiety symptoms during the COVID-19 outbreak compared to various control conditions. A total of 35 eligible studies with 5457 participants were included. The meta-analysis results showed that psychosocial and behavioral interventions had statistically significant moderate effects on depression [SMD =  − 0.73, 95% CI (− 1.01, − 0.45), *I*^2^ = 90%] and large effects on anxiety [SMD =  − 0.90, 95% CI (− 1.19, − 0.60), *I*^2^ = 92%], especially in the general population and COVID-19 survivors. Exercise and cognitive behavioral therapy were found to be the most effective treatments with moderate-to-large effect size for depression and anxiety during the outbreak of COVID-19. We also found the internet-based approach could also achieve almost equally significant effects on depression and anxiety compared with face-to-face traditional approach. Our findings suggest that cognitive behavioral therapy and physical exercise intervention are significantly effective for depression and anxiety related to the COVID-19 pandemic regardless of the delivery modes, and gender differences should be taken into consideration for better implementation of interventions in clinical and community practice.

## Introduction

In December 2019, the Coronavirus Disease 2019 (COVID-19) outbreak in China started to spread worldwide. It is a highly contagious respiratory pandemic with direct impacts on the physical health in patients with COVID-19, and a significant psychological burden for the general population^1,2^. Immediate contributing factors to mental health include fear of SARS-CoV-2 infection and related social stigma, as well as deteriorating social determinants such as socio-economic stressors^3,4^. To slow down the spread of COVID-19, many preventive measures, such as social distance and quarantine policy, have been adopted by governments around the world. These infection control measures are effective to prevent the escalation of public health emergencies. However, prolonged social distancing and home confinement have substantial adverse effects on mental health during the pandemic and long after^5^. Recently, an epidemiological study showed that the prevalence of depressive and anxiety disorders was exacerbated during the COVID-19 pandemic, with a predicted additional 53.2 and 76.2 million cases of major depression and anxiety disorder, respectively^6^. Timely and cost-effective interventions for mental wellbeing in public is highly recommended due to the unprecedented impact of COVID-19 pandemic.

Given that pharmacological interventions focusing on biological factors have been demonstrated to have limited effects and values on subclinical symptoms and many of these treatments have biobehavioral and clinical side effects, several prominent international institutions including the World Health Organization have suggested prioritizing psychosocial and behavioral interventions to reduce the global burden of mental health^7^. Psychosocial intervention puts emphasis on psychological or social factors instead of biological factors with a view to promoting mental wellbeing and functioning^8,9^. According to the UK Department of Health, psychosocial intervention therefore includes Health Education, interventions with a focus on social aspects, such as Social Support and Networking^10^. Behavioral intervention is also used to contrast with biomedical or pharmacological interventions, which is designed to identify behaviors or using behavioral approaches to restructure maladaptive behaviors^11,12^. These interventions involve action-based behavioral interventions such as physical exercise, and well-recognized psycho-behavioral modalities encompassing cognitive, behavioral, and mind–body exercise. Substantial evidence demonstrated that both psychosocial and behavioral interventions have beneficial effects on depression and anxiety. Inspired by these previous studies, psychosocial and behavioral interventions have been increasingly used to improve depressive and anxiety symptoms during the COVID-19 pandemic.

Numerous clinical trials and observational studies are underway around the world investigating the role of various psychosocial and behavioral interventions on mental health in various populations. However, the clinical evidence on the effectiveness of these interventions is scattered and heterogeneous, and therefore inconclusive due to use of different methodologies and multiple outcome measures across various settings. In addition, both face-to-face and internet-based approaches were used to deliver interventions during the pandemic and there was no comparison of the effectiveness of the different approaches. To address these issues, we performed a systematic review and meta-analysis of published randomized controlled trials (RCTs) to examine the pooling therapeutic effectiveness of psychosocial and behavioral interventions using the robust effect size estimation meta-analytic technique, as well as the effectiveness of different delivery ways, on mental health in individuals with depressive and anxiety symptoms during the COVID-19 pandemic.

## Methods

The systematic review and meta-analysis was carried out in accordance with the Preferred Reporting Items for Systematic Review and Meta-Analysis (PRISMA) guidelines^13^. A PRISMA checklist is provided in Supplementary Table 1. The registration number in PROSPERO is CRD42022303600.

### Search strategy

We conducted a comprehensive search of all articles published between December 2019 and February 2022 in the following four English and two Chinese electronic databases: PubMed, PsycINFO, Scopus, Web of Science, CNKI, and Wanfang Data. The search strategy is presented in Supplementary Table 2. Additionally, manual searches of the references list of all included papers were conducted to include any other eligible studies.

### Inclusion criteria

The selection criteria were based on the Population, Intervention, Comparison, Outcome, Settings (PICOS) model^14^. Studies that met the following criteria were included in the systematic review and meta-analysis.

*Participants*: Individuals who reported depression or anxiety symptoms during the COVID-19 pandemic were eligible. Any studies that included patients with psychiatric diagnosis were excluded.

*Interventions*: Psychosocial and behavioral treatments delivered through either traditional face-to-face or internet-based approaches. The psychosocial and behavioral interventions were categorized into five non-exclusive types based on the description of each included study: exercise, CBT, psychosocial education, mindfulness-based intervention, and multiple-component interventions. There was no limit regarding the frequency and duration of psychosocial and behavioral interventions.

*Exercise* refers to any planned, structured, and purposeful bodily activity that maintains or enhances physical fitness and mental health and wellness. *CBT* is structured psychotherapy that highlights the identification and correction of maladaptive thought patterns and behaviors to reduce depressive and anxiety symptoms and promotes psychological adjustment through goal-oriented intervention. *Psychosocial Education* varies in the amount of psychosocial information or coping instruction and behavioral training provided in each study, but emphasizes on knowledge and coping skills of depression and anxiety during the COVID-19 pandemic. *Mindfulness-based Intervention (MBI)* consists of the basic components proposed by Crane and colleagues including present-moment focus and decentering, developing greater attentional and behavioral self-regulation, and engaging the participant in sustained mindfulness meditation practice^15^. *Multiple-component interventions* are interventions with two or more different types of psychosocial and behavioral interventions mentioned above were employed in each intervention group by the included study.

*Comparison*: Studies involving at least a treatment group and a comparison group. Both active and non-active control groups were eligible as comparison groups.

*Outcomes*: Depressive and anxiety symptoms measured and reported in the original studies as primary or secondary outcomes using standardized self-reported scales or clinical diagnostic scales. Studies were excluded if there was no available data to calculate the pre-post change scores for depression and anxiety, or the information could not be retrieved from the authors.

*Settings*: Only RCTs conducted during the outbreak of COVID-19 with the sample recruitment after Dec 2019 were eligible. Studies such as quasi-RCTs, pilot trials, case–control studies, and case reports were excluded. All studies were published in peer-reviewed journals.

### Study selection and data extraction

Two review authors (JH and WS) individually screened the title and abstracts of all the eligible articles after removing duplicates. The full-text versions of the selected studies were then screened to determine whether they met our inclusion criteria. The data from all included studies were extracted and checked using a standard data extraction form. The following information was recorded in our review: study source, participant characteristics, sample size, details of the interventions, psychological status outcomes, and dropout rate. The relevant corresponding authors were contacted for any missing data or unreported data in the articles.

Several variables that potentially influenced the association between the psychosocial and behavioral interventions and mental state were coded. First, if the mean age and median age of participants were unavailable, the midpoint of the reported age range of participants was used. Second, the treatment was coded as “multiple interventions” whenever two or more psychosocial and/or behavioral interventions were conducted. In addition, we coded two studies as “multiple locations”, as they recruited participants from two or more countries. Lastly, the severity of participant’s depressive and anxiety states in each study was coded as “mild-to-moderate levels” and “severe levels” based on the criteria of the various standardized self-report scales used at the baseline assessment.

### Quality assessment

Two reviewers (JH and WS) evaluated the internal quality of the eligible studies based on the revised Cochrane Risk of Bias assessment tool (RoB 2.0)^16^. The RoB 2.0 tool consists of five domains for randomized trials: (1) bias arising from the randomization process, (2) bias due to deviations from the intended interventions, (3) bias due to missing outcome data, (4) bias in measuring the outcome, and (5) bias in selecting the reported results. Overall, each individual study was judged to have low risk of bias, some concerns, or high risk of bias.

All of the above procedures were conducted by the two independent review authors and any discrepancies were resolved in a consensus meeting with the third author (JL).

### Data analysis

All statistical analyses were performed using the "metafor" package in R version 4.1.0. In our meta-analysis, the standardized mean difference (SMD) of the primary outcome measure was used to assess the effectiveness of psychosocial and behavioral interventions on depressive and anxiety symptoms. Hedges’ g was used for the effect size estimation, with values of 0.2 to 0.5, 0.5 to 0.8, and above 0.8 corresponding to small, moderate, and large effect sizes, respectively^17^. Knapp-Hartung adjustment was used to estimate the 95% confidence interval (95% CI) to control the risk of false positives^18^. Cochran Q test was used to evaluate statistical heterogeneity, with *p* < 0.10 suggesting the presence of significant statistical heterogeneity^19^. The *I*^*2*^ statistic was used to assess the severity of heterogeneity, which was considered to be significant when *I*^2^ ≥ 50%^20^. The random-effects model was adopted to estimate the pooled effect sizes depending on the statistical significance of the *I*^2^ index^21^. In addition, the mean difference (MD) of the outcomes was extracted from each included study to estimate the real treatment effect of the intervention group vs the control group.

Subgroup meta-analyses were conducted to explore the heterogeneity of the effect sizes reported in each study according to the characteristic features of participants and interventions. To investigate potential factors associated with the effects, univariate and multiple meta-regression analyses were performed using mean age of participants, percentage of females, frequency of interventions, location where the research was conducted, and the type of control group as covariates. Sensitivity analyses were undertaken in the main meta-analysis by excluding studies with high risk of bias. Case-by-case sensitivity analyses were also performed by excluding each study in turn to determine if there were any outliers that might significantly affect the heterogeneity.

Begg's funnel plot^22^ and Egger's regression asymmetry test^23^ were used to interpret the presence of publication bias to verify the validity of all included studies. In addition, the trim-and-fill method was conducted in the main meta-analysis to remove non-symmetric effect sizes from the positive side of the funnel plot, and then a subsequent funnel plot with imputed values was regenerated to estimate an unbiased distribution^24^. Finally, a classic fail-safe analysis was performed to calculate the number of negative studies that would be necessary to decrease the combined effect sizes to a non-significant level (i.e., *p* > 0.05). A higher fail-safe N value suggested the pooled effect sizes of the meta-analysis would be more robust if the value exceeded Rosenthal’s recommended tolerance value of 5* k* + 10 (where *k* is the number of effect sizes)^25^.

## Results

### Study selection and characteristics

**Figure 1:**
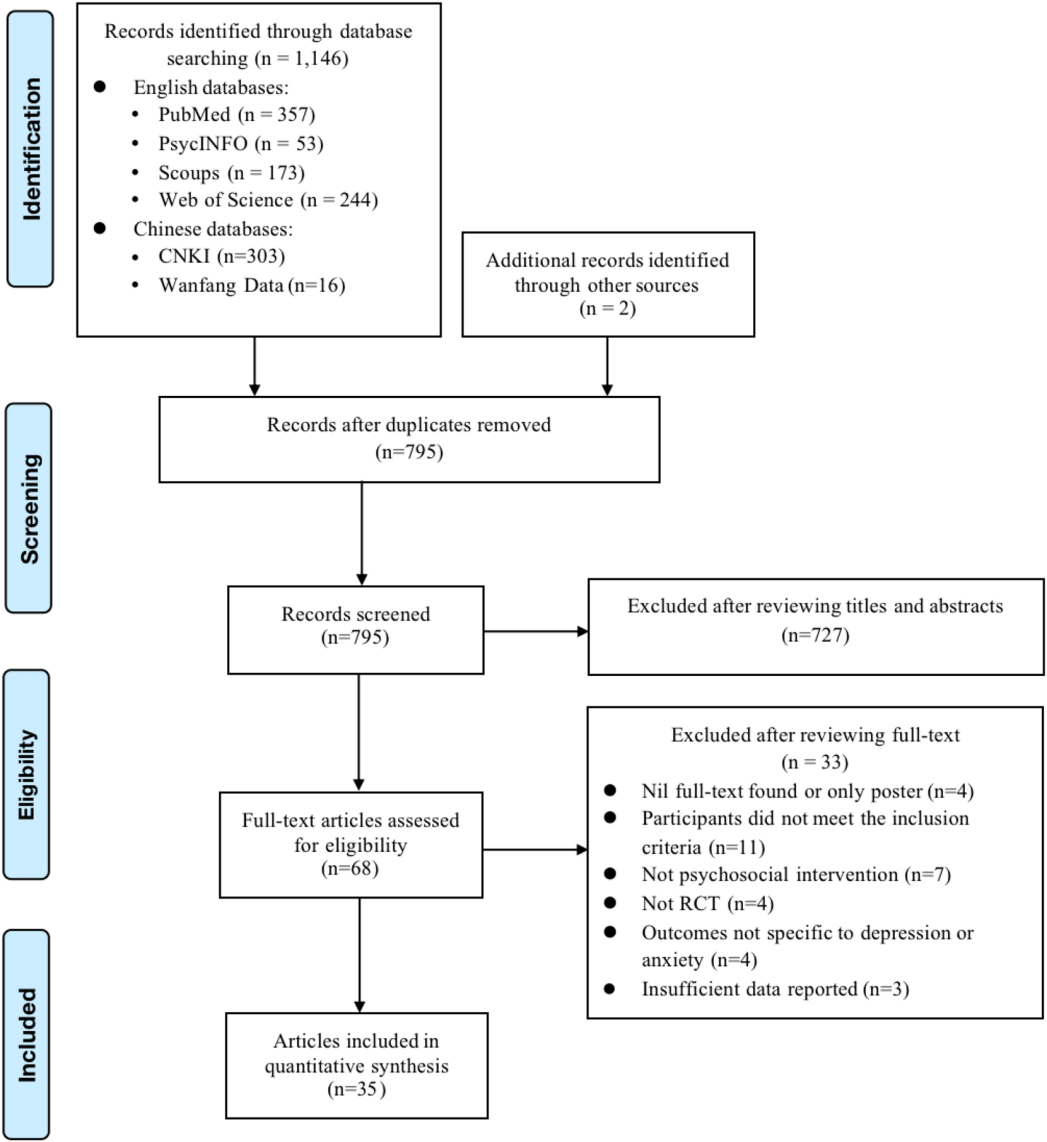
PRISMA flow diagram of the search results.

Figure 1 illustrates the PRISMA flow diagram of the study retrieval process. A total of 1,146 studies were identified from four English and two Chinese databases. Two additional studies were manually added after reviewing the references list of all the included papers. The final sample included 35 RCTs with 5457 participants (3583 females and 1685 males) from five regions: America (1 study), Canada (2 studies), China (19 studies), Europe (6 studies), West Asia (5 studies), and multiple countries (2 studies). Among the studies included in the quantitative analyses, 14 examined the effects of interventions on people with COVID-19, four on medical professionals, and 17 on the general population. The mean age of the included participants ranged from 14.53 to 73.03 years, with a median age of 38.46 years. The psychosocial and behavioral interventions were categorized into five different types based on the descriptions provided by each study: exercise, CBT, psychosocial education, mindfulness-based intervention, and multiple-component interventions.

All studies used self-report questionnaires to evaluate the depressive and anxiety symptoms of the participants. Twenty-one studies were reported as mild-to-moderate depressive symptoms and the remaining studies were severe based on the mean score of primary measure of each included study. Seventeen studies were assessed as mild-to-moderate anxiety symptoms and sixteen studies were severe. The intervention sessions ranged from 2 to 42 sessions and the duration of the interventions ranged from 1 to 12 weeks. An active comparator was used in 12 studies and a treat-as-usual control group was used in the remaining 23 studies. Specifically, 27 of the 35 studies investigated the effectiveness of psychosocial and behavioral interventions on depression, and 33 studies focused on psychosocial and behavioral interventions for anxiety. Table 1 provides the characteristics of included RCTs.

**Table 1:** Characteristics of the included RCTs.

Author, year (Region)	Population group	Sample size	Age mean (SD)	Gender (F/M)	Intervention type	Frequency, duration	Psychological status measured	Dropout rate
Beauchamp et al.^26^ (Canada)	Older adults	241	73.03 (5.42)	I1: 65/15	Internet-based physical exercise	50 to 60 min/time, > 3 times/week, 12 weeks	CES-D	15/239 (12.01%)
			I1: 73.0 (5.0)	I2: 64/18				I1: 7/80 (8.75%)
								I2: 5/82 (6.10%)
			I2: 74.1 (6.2)	C: 59/20				C: 3/79 (3.80%)
			C: 72.0 (4.8)					
Borrega-Mouquinho et al.^27^ (Spain)	General population	67	26.13 (7.17)	I: 21/15	Internet-based HIIT	40 min/time 6 days/week, 6 weeks	BDI-13, STAI, PSS	14/67 (21%)
								I: 8/36 (22%)
			I: 25.22 (5.23)					C: 6/31(19%)
			C: 27.19 (8.88)	C: 24/7				
Chen et al.^28^ (China)	Older adults with COVID-19	29	I: 67.6 (11.2)	I: 7/7	Baduanjin on bed	20 to 30 min/time, > 10 times/week, 3 weeks	SAS, SDS	3/32 (9.38%)
			C: 68.5 (10.8)	C: 9/6				I: 2/16 (%)
								C: 1/16 (%)
Cozzolino et al..^29^ (Italy)	Students	310	28.73 (9.16)	241/69	Internet-based mind–body practice	15 min/time, 1 time/week, 4 weeks	STAI	44/310 (14.19%)
								I: 11/155 (7%)
								C: 33/155 (21%)
Cui et al.^30^ (China)	General population	148	I: 35.88 (8.30)	I: 49/25	Internet-based mindful stress-reduction therapy	45 to 60 min/time, 2 times/week, 2 weeks	PHQ-9, GAD-7	0%
			C: 36.69 (9.17)	C: 41/33				
Egan et al.^31^ (Multiple countries)	General population	225	37.79 (14.02)	I: 96/14 (Non-binary: 1; Preferred not to say: 1)	Internet-based unguided low intensity CBT	7 times/week, 1 week	PHQ-9, GAD-7	53/225 (23.11%)
			I: 36.88(13.33)					
			C: 38.69(14.68)	C: 95/17 (Non-binary: 1)				I: 29/112 (25%)
								C: 24/113 (21.24%)
Fan et al.^32^ (China)	Patients with COVID-19	111	46.38 (12.34)	I: 34/22	NET + personalized psychological treatment	90 to 120 min/time, 1 to 2 times/week, 8 weeks	SDS, SAS, PSQI	0%
			I: 46.16 (12.01)	C: 35/20				
			C: 46.60 (12.79)					
Fiol-DeRoque et al.^33^ (Spain)	Health care workers	482	41.37 (10.4)	I: 210/38	Internet-based psycho-educational and mindfulness	2 weeks	DASS-21, ISI	46/482 (9.50%)
			I: 42.07 (11.0)					I: 27/248 (11%)
			C: 40.62 (9.6)	I: 210/38				C: 19/234 (8%)
Ghazanfarpour et al.^34^ (Iran)	Medical workers	95	N/A	I: 7/44	Internet-based tele-counseling	45 to 90 min/time, 7 times, 7 consecutive days	HADS, SHAI	8/95 (8.16%)
				C: 8/36				I: 2/51 (3.92%)
								C: 6/44 (13.64%)
Gu et al.^35^ (China)	Patients with COVID-19	63	I: 40.85 (13.14)	I: 14/19	Internet-based mindfulness-based stress reduction	20 to 30 min/ time, 5 times/week, 4 weeks	SAS, SDS	7/70 (10%)
			C: 40.23 (13.88)	C: 15/15				I: 2/35 (%)
								C: 5/35 (%)
He et al.^36^ (China)	Older adults	104	I: 67.42 (3.19)	I: 25/27	Psychotherapy	N/A	SCL-90	0%
			C: 67.55 (4.20)	C: 24/28				
Kam et al.^37^ (America)	General population	62	I: 30.91 (SE = 0.37)	I: 27/5	Internet-based mindfulness training	> 10 min/day, 10 days	PROMIS	0%
			C: 28.73 (SE = 0.10)	C: 25/5				
Kong et al.^38^ (China)	Patients with COVID-19	26	N/A	N/A	Psychological-behavioral intervention	20 min breathing exercise + 15 min psychological support intervention, 1 time/day, 10 days	HADS	0%
Latino et al.^39^ (Italy)	High-school students	30	14.53 (0.5)	I: 8/7	Internet-based physical activity	60 min/time, 2 times/week, 8 weeks	QAS-anxiety	0%
				C: 5/10				
Li^40^ (China)	Nurses	96	I: 29.14 (4.32)	I: 48/0	Mindfulness relaxation	N/A	SAS, SDS, PSQI	0%
			C: 29.43 (4.27)	C: 48/0				
Li et al.^41^ (China)	Patients with COVID-19	93	I: 48.3 (12.2)	I: 34/13	CBT	30 min/time, 1 time/day	DASS-21	1/94 (1.06%)
			C: 47.1 (10.6)	C: 26/20				C: 1/47 (2.12%)
Liang et al.^42^ (China)	Medical students	52	I: 20.73 (1.87)	I: 16/10	Dialectical behavior group therapy	90 min/time, 2 times/week, 4 weeks	PHQ-9, GAD-7, PSS-10	0%
			C: 20.62 (1.79)	C: 16/10				
Liu et al.^43^ (China)	Patients with COVID-19	252	I: 43.76 (14.31)	I: 56/70	Internet-based CBT	> 10 min/day, 1 week	HAMA, HAMD, SDS, SAS, AIS	0%
			C: 41.52 (11.51)	C: 46/80				
Öner Cengiz et al.^44^ (Turkey)	Patients with COVID-19	44	51.64 (14.16)	I: 12/10	Deep breathing exercises	5 to 10 time/hour	BAI	6/50 (12%)
			I: 49.18 (13.50)	C: 11/11				I: 3/25 (12%)
			C: 54.09 (14.68)					C: 3/25 (12%)
Özlü et al.^45^ (Turkey)	Patients with COVID-19	67	I: 36.48 (11.63)	I: 12/21	Progressive muscle relaxation exercise	20 to 30 min/time, 2 times/day, 5 days	STAI	6/73 (8.22%)
								I: 3/36 (8.33%)
			I: 36.48 (11.63)	I: 12/21				C: 3/37 (8.11%)
Pan et al.^46^ (China)	Suspected Patients with COVID-19	64	I: 39.13 (14.97)	26/38	Psychological treatment	N/A	SAS, SDS	0%
			C: 37.06 (14.08)					
Parizad et al.^47^ (Iran)	Patients with COVID-19	110	I: 43.14 (12.22)	I: 25/30	Guided imagery (CBT)	30 min/time, 2 times/day, 5 consecutive days	STAI	0%
			C: 37.32 (11.12)	C: 23/32				
Puterman et al.^48^ (Canada)	General population	334	40.3 (12.4)	I1: 71/11	Internet-based	20 min/time, 4 times/week, 6 weeks	CES-D	7/334 (2.10%)
								I1: 3/82 (3.66%)
			I1: 41.2 (12.7)	I2: 72/14	I1: HIIT			I2: 1/86 (1.16%)
								I3: 2/83 (2.41%)
			I2: 37.8 (12.3)	I3: 72/11	I1: HIIT			C: 1/83 (1.20%)
			I3: 41.1 (12.6)	C: 74/9	I3: HIIT + Yoga			
			I3: 41.1 (12.6)					
Shabahang et al.^49^ (Iran)	College students	150	24.7 (5.4)	77/73	Internet-based CBT	15 to 20 min/time, 3 times/week, 3 weeks	CVAQ, SHAI, ASI-3	2/152 (1.32%)
								I: 1/76 (1.32%)
								C: 1/76 (1.32%)
Solianik et al.^50^ (Lithuania)	Older adults	30	67.0 (5.9)	I: 13/2	Tai chi	60 min/time, 2 times/week, 10 weeks	PSS-10, HADS	0%
				C: 13/2				
Wahlund et al.^51^ (Sweden)	General population	670	I: 45 (13)	I: 277/58	Internet-based CBT	3 weeks	GAD-7, MADRS-S, ISI	71/670 (10.60%)
								I: 50/335 (14.93%)
			C: 47 (14)	C: 270/65				C: 21/335 (6.27%)
Wang et al.^52^ (China)	Patients with fever	110	I: 33.8 (13.2)	I: 23/32	Multiple psychological treatment	10 to 20 min/time for every two hours	SAS	0%
			C: 32 (11.6)	C: 33/22				
Wang et al.^53^ (China)	Patients with COVID-19	56	I: 49.3 (17.1)	I: 13/15	Psychological treatment	N/A	SAS, SDS	0%
			C: 50.2 (16.8)	C: 14/14				
Wilke et al.^54^ (Multiple countries)	General population	763	32.8 (12.6)	I: 270/115	Internet-based physical exercise	30 to 60 min/time, 5 times/week, 4 weeks	GAD-7	413/763 (54%)
			I: 32.9 (13.1)	C: 253/122				I: 197/386 (51%)
			C: 32.6 (12.1)					C: 215/377 (57%)
Yang et al.^55^ (China)	College students	104	I: 18.72 (0.66)	I: 29/24	Internet-based mindfulness relaxation	30 min/time, once every other day, 10 days	DASS-21, PSQI	0%
			C: 18.49 (0.81)	C: 26/25				
Zhang et al.^56^ (China)	Adolescents	153	I: 15.7 (2.05)	I: 35/41	Psychological counseling and outdoor exercises	1 h/time, 1 time/week, 8 weeks	SAS, SDS, PSQI	7/160 (4.38%)
			C: 15.9 (1.07)	C: 38/39				I: 4/80 (5.00%)
								C: 3/80 (3.75%)
Zhang^57^ (China)	College students	90	N/A	N/A	Internet-based Qigong	60 min /time, 3 months	SAS, SDS, PSQI	0%
Zhang & Rao^58^ (China)	Patients with COVID-19	28	I: 49.87 (4.23)	I: 6/8	Qigong	60 min/time, 2 times/day, 3 days/week	SAS, SDS	0%
			C: 49.89 (4.22)	C: 5/9				
Zhou et al.^59^ (China)	Nurses	118	I: 31.0 (4.4)	I: 59/1	Internet-based CBT	7 time/week, 6 weeks	GAD-7, ISI, PHQ-9, PSQI	0%
			C: 29.6 (4.5)	C: 57/1				
Zhu et al.^60^ (China)	Patients with COVID-19	80	I: 36.23 (10.5)	I: 18/22	CBT	9 days	SAS, SDS	0%
			C: 36.4 (13.05)	C: 16/24				

*CBT* cognitive behavioral therapy, *HIIT* high-intensity interval training, *NET* narrative exposure therapy, AIS Athens Insomnia Scale, *BAI* beck anxiety inventory, *BDI* beck depression inventory, *BDI-II* beck depression inventory-II, *CVAQ* COVID-19 anxiety questionnaire, *CES-D* center for epidemiologic studies depression scale, *DASS-21* depression, Anxiety, and Stress Scale-21 Items, *GAD-7* general anxiety disorder-7, *HADS* hospital anxiety and depression scale, *HAMA* Hamilton rating scale for anxiety, *HAMD* Hamilton rating scale for depression, *ISI* insomnia severity index, *MADSR* Montgomery-Asberg depression rating scale, *PHQ-9* patient health questionnaire-9, *PSQI* Pittsburgh sleep quality index, *PSS-10* perceived stress scale, *PROMIS* patient-reported outcomes measurement information system, *QAS* study approach Questionnaire (QAS)—anxiety, *SAS* self-rating anxiety scale, *SCL-90* symptom checklist-90, *SDS* self-rating depression scale, *SHAI* short health anxiety inventory, *STAI* state-trait anxiety inventory.

### Risk of bias

Overall, three studies were considered as having high risk of bias, 19 studies had some concerns, and 13 studies had low risk of bias. For bias arising from the randomization process, all included studies were described as randomized, with 17 studies reporting an adequate random sequence generation method, and the remaining 18 studies reporting random grouping, but gave no details of allocation concealment, which may raise some concerns. For bias due to missing outcome data, three studies were rated as having a high risk of bias. All studies were classified to be at low risk of bias in the remaining three domains. Supplementary Table 3 provides the risk of bias for all included studies.

### The effects of interventions on depression

**Figure 2:**
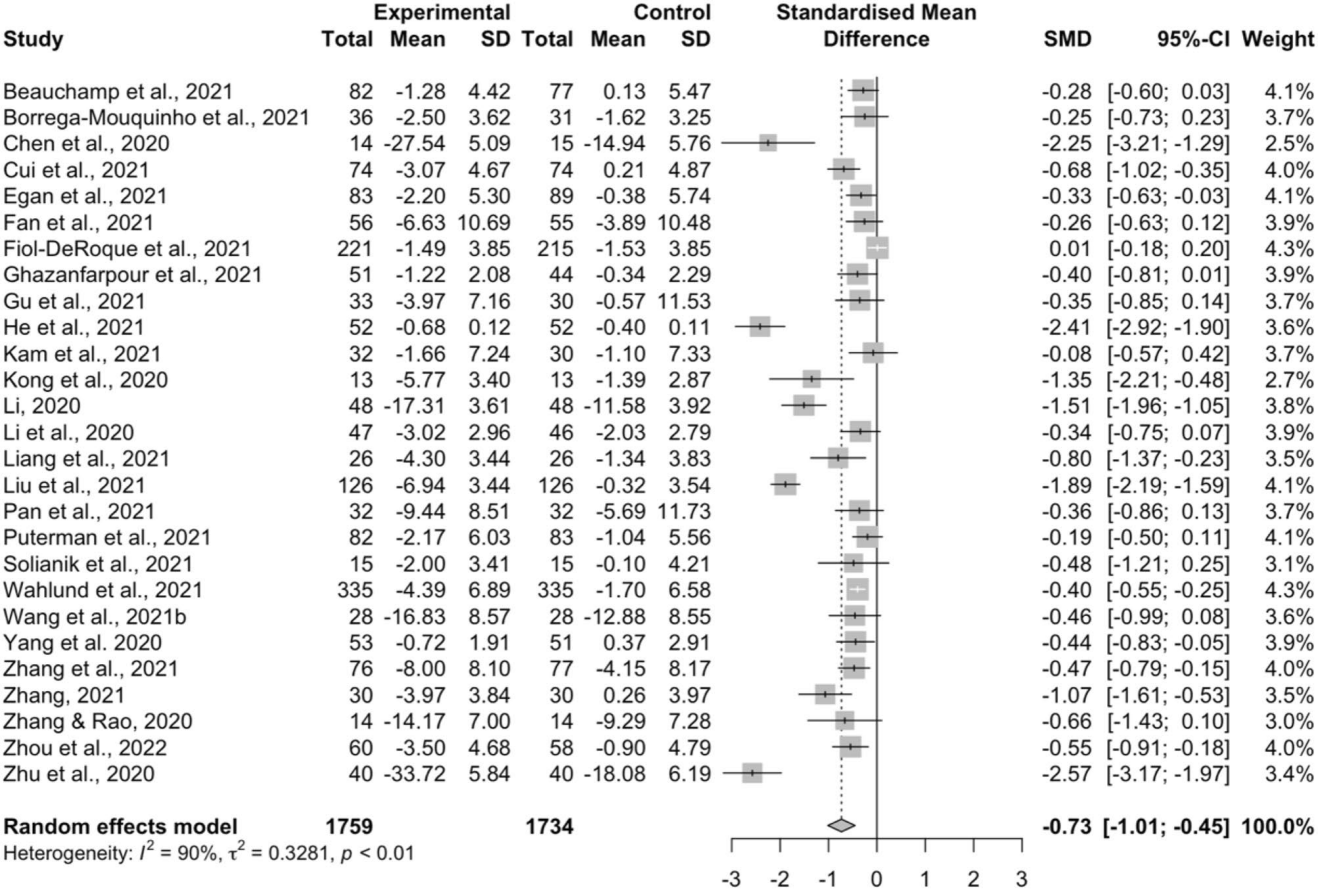
Forest plot depicting the effects of psychosocial and behavioral interventions on depression.

Figure 2 shows the overall effect sizes of all interventions for depression. The results of 27 studies were pooled, which revealed that psychosocial and behavioral interventions significantly reduced depression levels during the COVID-19 pandemic compared to control groups [SMD =  − 0.73, 95% CI (− 1.01, − 0.45), *I*^2^ = 90%] with a median effect size of − 0.46 (range =  − 2.57 to 0.01). In addition, Supplementary Table 6 presents the MD of each included study regarding the treatment effect of such interventions on depressive symptoms.

Full details of all the subgroup analysis results for depression are given in Supplementary Table 4. Our results indicated that all interventions psychosocial and behavioral interventions significantly reduced depressive symptoms in participants aged between 15.70 and 49.88 years [SMD =  − 0.64, 95% CI (− 0.91, − 0.38), *I*^2^ = 89%], the general population [SMD =  − 0.58, 95% CI (− 0.93, − 0.23), *I*^2^ = 84%], and COVID-19 survivors [SMD =  − 1.02, 95% CI (− 1.66, − 0.39), *I*^2^ = 92%]. However, the subgroup analysis also showed that psychosocial and behavioral interventions did not significantly improve depression symptoms in older participants [SMD =  − 1.34, 95% CI (− 3.14, 0.47), *I*^2^ = 95%] and medical professionals [SMD =  − 0.59, 95% CI (− 1.61, 0.43), *I*^2^ = 92%]. Among the five intervention types mentioned above, only two types, exercise and CBT, showed significant improvements on depression with a moderate effect size [SMD =  − 0.62, 95% CI (− 1.21, − 0.03), *I*^2^ = 74%] and a large effect size [SMD =  − 0.87, 95% CI (− 1.58, − 0.16), *I*^2^ = 95%], respectively. Subgroup analysis also indicated that only studies conducted in China [SMD =  − 1.00, 95% CI (− 1.38, − 0.61), *I*^2^ = 90%] and multiple countries [SMD =  − 0.33, 95% CI (− 0.63, − 0.03), *k* = 1] showed significant improvements on depressive symptoms with psychosocial and behavioral interventions. Furthermore, compared with the control groups, both traditional face-to-face [SMD =  − 0.97, 95% CI (− 1.47, − 0.48), *I*^2^ = 89%] and internet-based [SMD =  − 0.52, 95% CI (− 0.82, − 0.22), *I*^2^ = 90%] delivery approaches yielded statistically significant benefits on depression.

### The effects of interventions on anxiety

**Figure 3:**
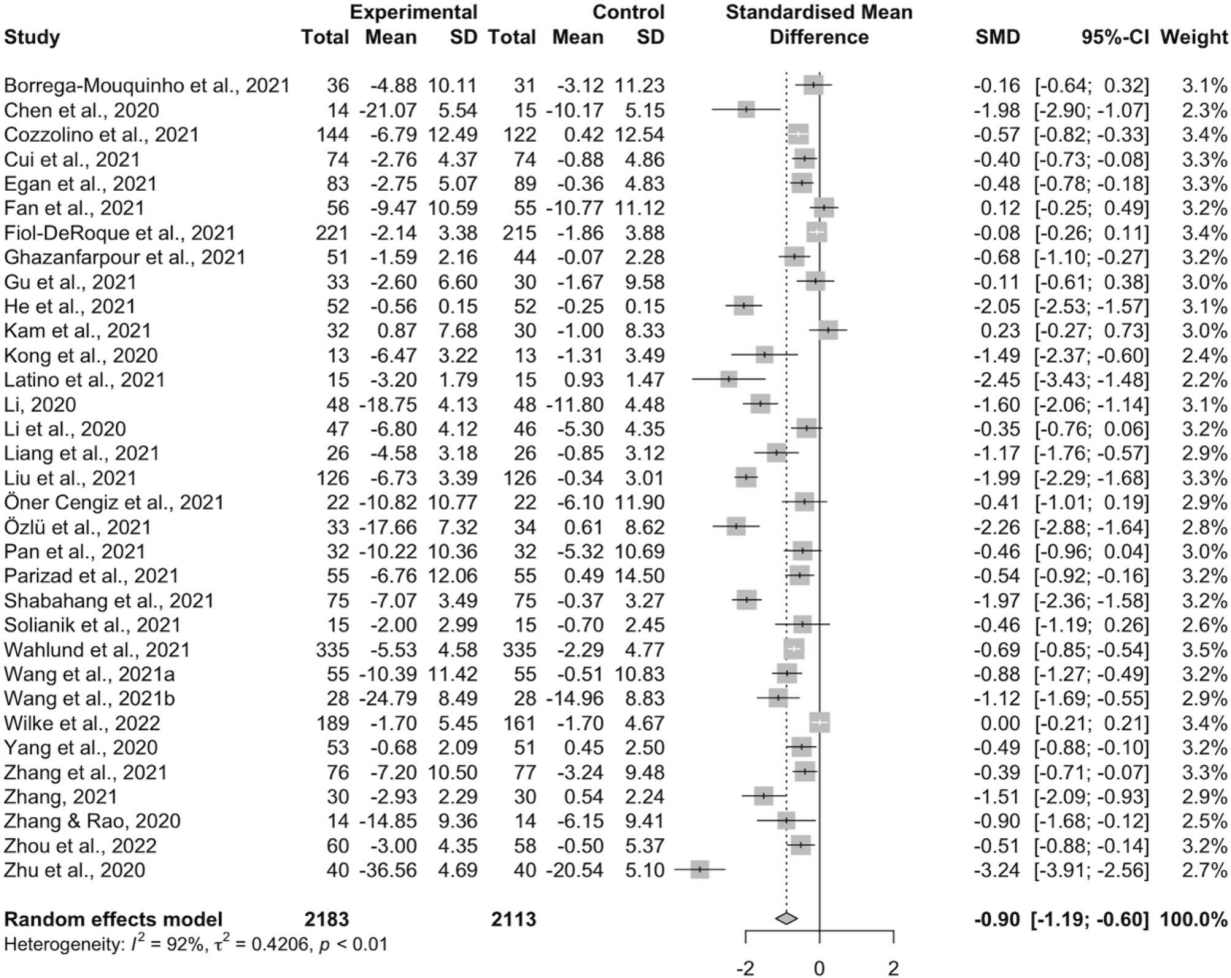
Forest plot depicting the effects of psychosocial and behavioral interventions on anxiety.

Figure 3 shows the overall effect sizes for all interventions on anxiety. The results of 33 studies were pooled, which revealed all interventions were able to significantly decrease anxiety levels during the pandemic compared to control groups [SMD =  − 0.90, 95% CI (− 1.19, − 0.60), *I*^2^ = 92%] with a median effect size of − 0.57 (range =  − 3.24 to 0.23). The MD for the beneficial effect of psychosocial and behavioral interventions on anxiety symptoms in each included study is provided in Supplementary Table 6.

Full details of the subgroup analysis results with heterogeneity statistics for anxiety are presented in Supplementary Table 5. Our analyses revealed that allpsychosocial and behavioral interventions achieved a statistically notable drop in anxiety level in participants aged below 50 years old [SMD =  − 0.84, 95% CI (− 1.14, − 0.54), *I*^2^ = 92%], in the general population [SMD =  − 0.79, 95% CI (− 1.21, − 0.36), *I*^2^ = 90%], and patients with COVID-19 [SMD =  − 1.09, 95% CI (− 1.64, − 0.54), *I*^2^ = 93%]. In contrast, the subgroup analysis showed no significant decreases in anxiety level in older participants [SMD =  − 1.50, 95% CI (− 3.74, 0.73), *I*^2^ = 85%] and medical professionals [SMD =  − 0.69, 95% CI (− 1.71, 0.32), *I*^2^ = 92%]. Among the five intervention types mentioned above, only two types, exercise and CBT, showed significant improvements on anxiety, with both eliciting large effect sizes [SMD =  − 1.00, 95% CI (− 1.64, − 0.36), *I*^2^ = 90%] and [SMD =  − 1.05, 95% CI (− 1.77, − 0.34), *I*^2^ = 95%], respectively. In addition, significant benefits of psychosocial and behavioral interventions on anxiety symptoms were only found in China [SMD =  − 1.05, 95% CI (− 1.45, − 0.65), *I*^2^ = 92%] and West Asia [SMD =  − 1.16, 95% CI (− 2.24, − 0.09), *I*^2^ = 92%]. For delivery approaches, both traditional face-to-face [SMD =  − 1.02, 95% CI (− 1.47, − 0.58), *I*^2^ = 90%] and internet-based [SMD =  − 0.76, 95% CI (− 1.17, − 0.35), *I*^2^ = 94%] interventions showed significant effects on reducing anxiety compared with the control group.

### Meta-regression

In the univariate meta-regression, the results revealed that participant’s age, gender difference, length of intervention, the location where the research was conducted, type of control group, and severity of baseline depressive and anxiety states were not significantly related to the effects of psychosocial and behavioral interventions on depression and anxiety. Multiple meta-regression analyses suggested a significant gender × type of intervention effect on anxiety for CBT [β = 0.03, 95% CI (0.00, 0.06), *p* = 0.044] and exercise [β = 0.04, 95% CI (0.00, 0.07), *p* = 0.032] compared with mindfulness-based intervention, with a superior effect in males. Supplementary Tables 7–9 summarize the univariate and multiple meta-regression analyses of the associated factors on depression and anxiety, respectively.

### Publication bias

Three different tests were used in our meta-analysis to evaluate publication bias. Egger’s regression test showed there was significant publication bias in both depression (*p* = 0.030) and anxiety (*p* = 0.014) studies. However, the asymmetry of funnel plot was not prominent (Supplementary Figs. 1 and 2), with no significant changes in the bias-adjusted effects for depression [SMD = − 0.66, 95% CI (− 0.98, − 0.34), adjusted studies = 1], but the trim-and-fill effect size was slightly decreased and significant for anxiety [SMD = − 0.43, 95% CI (− 0.80, − 0.07), adjusted studies = 10]. Furthermore, the fail-safe N value required to nullify the overall effect sizes of psychosocial and behavioral interventions for depression (N = 2761) and anxiety (N = 5771) showed no significant publication bias. Overall, our results were verified to be relatively robust.

### Sensitivity analysis

As we detected severe heterogeneity in the included studies, we conducted further sensitivity analyses to evaluate the impact of the quality of the studies on the findings. No outlier was found that would exert a significant impact on heterogeneity when excluding studies using a case-by-case approach. After excluding four studies with high risk of bias, there were still no significant differences in the overall effects on depression and anxiety. When removing several studies without information of intervention duration and frequency, the heterogeneity of the pooled effects of psychosocial and behavioral interventions on depression was dramatically reduced, but the overall effects of all types of intervention remained significant [SMD = − 0.44, 95% CI (− 0.63, − 0.26), *I*^2^ = 44%] (Supplementary Fig. 3). Repeating the analysis, the heterogeneity of the included studies on anxiety were unchanged, with the overall effects remaining significant [SMD = − 0.73, 95% CI (− 1.11, − 0.36), *I*^2^ = 89%].

## Discussion

This is an up-to-date review of 35 RCTs on psychosocial and behavioral interventions for depression and anxiety involving 5457 participants. The cumulative evidence from our meta-analysis suggests that psychosocial and behavioral interventions during the COVID-19 pandemic were associated with significant reductions in depressive and anxiety symptoms, especially for COVID-19 survivors and the general population. Exercise and CBT are the two types of psychosocial and behavioral treatments that were found to have the most benefits on psychological wellbeing. To our knowledge, this is the first study to systematically identify and analyze the effects of psychosocial and behavioral interventions on depressive and anxiety symptoms in various populations during the COVID-19 pandemic.

In our review, we summarized five types of psychosocial and behavioral interventions. Exercise and CBT yielded significant moderate-to-large improvements in both depressive and anxiety symptoms, which were consistent with the findings from previous studies. A systematic review and meta-analysis of RCTs by Rebar et al.^61^ revealed that physical activity had small-to-medium effect sizes on depression and anxiety for non-clinical populations. In addition, Weitz and colleagues found CBT also had a moderate effect sizes on depression and anxiety symptoms^62^. Our finding demonstrated that exercise had similar effects with CBT on depression and anxiety, which strengthened the role of exercise in improving psychological well-being. It is suggested that these two well-established interventions should be considered in clinical practice to manage depressive and anxiety symptoms related to public health pandemic. In line with these findings, existing neurophysiological evidence indicated the underlying mechanisms of exercise and CBT on improving depression and anxiety levels act through positive neurobiology and neuroimmunology effects, such as the hypothalamic–pituitary–adrenal (HPA) axis and cortical-limbic pathways^63,64^. Taken together, the available literature confirms that exercise and CBT have beneficial effects that could be further promoted as a preventive and rehabilitative strategy to improve emotional-related symptoms during the pandemic. Nevertheless, previous reviews have shown that the other three types of interventions (mindfulness-based interventions, psychoeducation, and multiple interventions) can also elicit significantly positive effects on psychiatric distress^65–68^, although the findings of the current study do not support these earlier findings. However, it is important to note that the number of available studies in the subgroup meta-analysis was small, which may not achieve sufficient power to detect relatively subtle subgroup differences, despite the individual studies reporting significant effects for these three types of intervention.

We found that the subgroup analyses did not show significant improvements on depression and anxiety in older people. Before the outbreak of COVID-19, Pinquart and colleagues conducted a meta-analysis and found that psychosocial and behavioral interventions had significantly large effects on improving depressive and anxiety symptoms in older persons^69^. In contrast to previous findings, our results showed that older persons with depressive or anxiety symptoms did not benefit from psychosocial and behavioral interventions. One possible explanation is that the healthy older population generally tends to have higher resilience to emotional regulation compared with the younger population^70^. Recent case reports by Vahia et al.^71^ support the assumption that the mental health of healthy older persons were less negatively affected by COVID-19. Moreover, as the older population is at a higher risk of severe COVID-19 illness^72^, they may have had better control measures to mitigate infections, which would likely lead to reduced levels of depression and anxiety due to a sense of safety. However, these results should be interpreted with caution, as there were only four studies on older people included in our meta-analysis. The limited number of studies potentially leads to decreased statistical power to evaluate the beneficial effects. In addition, the subgroup analysis indicated that psychosocial and behavioral interventions only showed significant improvement on depressive symptoms in China and a study conducted in multiple regions. Meanwhile, the significant improvements on anxiety were found only in China and West Asia. However, it should be noted that the small number of included RCTs conducted in different regions, especially in some western countries, has limited the statistical power to detect the treatment effects and the results may not be readily generalizable. Readers should take careful consideration of this limitation when reading the results of this study.

Another notable finding is that psychosocial and behavioral intervention delivered in a face-to-face traditional approach and delivered using an internet-based approach both achieved significant effects on depression and anxiety, although the former approach had superior effects. The face-to-face format had large significant effects on both depression and anxiety, whereas the online intervention had small-to-moderate effects. However, several studies showed that these two treatment modes produced equivalent effects in treating psychological distress, although they emphasized that the face-to-face modality was probably not crucial for producing a large therapeutic effect as indicated in the previous literature^73–75^. A review by Johansson & Andersson^76^ discussed the potential factors that could affect the treatment effect of internet-based psychosocial and behavioral interventions, such as the degree of support provided and the role of targeted groups. Our results are not entirely consistent with the previous evidence, partially due to the lack of such factors associated with the effects of different intervention delivery modes. Future research should investigate the association of possible mediators and their effects on internet-based and face-to-face delivery modes to draw firm conclusions on whether they are equally effective. In addition to their beneficial effects, the availability, accessibility, and cost-effectiveness of these approaches on improving public mental health during the COVID-19 pandemic need to be assessed for future treatment strategies to help contain the disease. Nearly all psychiatric services have conducted infection control strategies to prevent the possibility of hospital-acquired infections, for instance, reducing outpatient appointments and placing greater constraints on admission to inpatient psychiatric units, which may make it challenging to access medications or receive in-person treatments, particularly for people who are in great need of psychological support^77^. Unfortunately, the extra demands on mental health services have also made it extremely difficult to meet the needs during the pandemic^78,79^. Even before the start of the COVID-19 outbreak, depression and anxiety were major contributors to the global health burden^80,81^. Thus, online delivery of psychosocial and behavioral interventions could be a feasible and promising approach for reducing depression and anxiety in general population. Individualized and tailored therapy can also be considered for different population groups according to their needs.

We also found a significant gender × intervention type interaction effect, in which females benefitted more from mindfulness-based intervention than CBT and exercise. This result also echoes the findings reported in previous studies that showed that gender-based divergent effects of psychosocial and behavioral interventions on anxiety. McRae et al.^82^ suggested that gender-based physiological differences were a key factor strongly associated with the gender-based divergent effects of interventions on anxiety. It has been widely reported that females and males have different responses to negative emotions. Johnson and Whisman^83^ reported that females tended to internalize their psychiatric distress by ruminating or engaging in self-critical behaviors, whereas males tended to externalize this by distracting themselves or engaging with the environment. This argument is supported by neuroimaging findings that showed negative emotions induced during working memory tasks led to the activation of emotion-related regions (amygdala and the orbitofrontal cortex) in females, whereas regions associated with cognitive control (prefrontal and superior parietal regions) remained more activated in males^84^. Our results also agreed with findings that mindfulness interventions were more effective in females by reducing negative emotional tendencies, whereas CBT and physical exercise were more effective in males by providing a better external coping strategy. To our knowledge, this pioneering study is first to show gender-based differences in psychosocial and behavioral interventions during the COVID-19 pandemic. Our results further highlight the importance of gender-specific and individualized interventions to improve psychological wellbeing during this public health crisis.

Psychosocial and behavioral interventions had shown significant improvements on depression and anxiety before the COVID-19 outbreak, however, several limitations have hindered the implementation of these interventions in general population. First, most existing evidence of the psychosocial and behavioral interventions focused on patients with psychiatric disorders or other chronic disease comorbidities. During the COVID-19 pandemic, depressive and anxiety symptoms appeared widely in general public^1^. Given that COVID-19 has spread worldwide, including the low- and middle-income countries where there are significant disparities in access to mental health services, it is important to ensure that evidence-based interventions are employed effectively to maximize the benefits of already overstretched resources. Our findings suggested that psychosocial and behavioral interventions demonstrated beneficial effects in the general population, particularly the younger people, as well as the COVID-19 survivors. Furthermore, we found that there was no obvious difference between traditional face-to-face and internet-based approaches. Both delivery modes achieved significant improvements in depression and anxiety. These evidences strengthened the potentials of psychosocial and behavioral interventions in depression and anxiety induced by public health crisis. We did not find significant improvements of these interventions in older adults and medical staff reported depression and anxiety in the sub-group analyses, which may be explained by the inefficient data included in the meta-analysis with only four studies in older people and in medical professionals. The limited number of studies potentially leads to decreased statistical power to evaluate the beneficial effects.

COVID-19 is the first pandemic in human history to simultaneously impact the global economy and health and disrupt multiple aspects of life for the majority of the world’s population. In the absence of a comprehensive global vaccination strategy or more effective biomedical therapies against this evolving virus, it is expected there will be periodic outbreaks in the coming years. Furthermore, increasing evidence suggests that the negative mental health impacts caused by the COVID-19 will be substantial and long-lasting, particularly among the vulnerable population^85^. Tackling this growing mental health burden will therefore be an urgent global challenge. Nevertheless, this also presents a historic opportunity for all countries and global agencies to cooperate on multiple and practical approaches to effectively address mental health issues due to public health emergencies such as the pandemic. The present systematic review and meta-analysis synthesizes the available RCTs to reveal that psychosocial and behavioral interventions, particularly exercise and CBT, are beneficial for public health crisis-related depression and anxiety. Our findings provide important implications for the promotion and implementation of these effective programs to improve coping strategy and mental wellbeing of the general population during the public health crisis.

### Limitations

The study has several limitations that need to be considered when interpreting the results. First, there was a moderate-to-high level of heterogeneity between the studies included in our meta-analysis. To investigate the potential sources of the heterogeneity of the treatment effects, we conducted a range of subgroup analyses and sensitivity analyses. The results should be interpreted with caution because of the limited number of RCTs in the subgroup analyses, which means the conclusions may not be readily generalizable. Besides, very few eligible studies from each country were identified in the search process, which limits the statistical power to detect differences in each region. Second, many clinical trials are still ongoing or under review as we write this article. This unpublished data could affect the magnitude of the observed effect sizes, although the statistical tests did not show a risk of publication bias. In addition, studies published in languages other than English and Chinese were not included in this review. Third, no study clearly defined that the depression and anxiety symptoms were induced specifically by the COVID-19 pandemic. It is important to differentiate the sources of mental health during the pandemic. Future studies should investigate the effects of the interventions on mental health induced by different events.

## Conclusions

Our systematic review and meta-analysis highlights the promising effects of psychosocial and behavioral interventions, particular CBT and exercise interventions, on depression and anxiety during the COVID-19 pandemic. Further research with large sample sizes with better methodological quality is needed to provide solid evidence for promoting these interventions for mental wellbeing during the pandemic and other public health crisis. Individuals who have experienced mental distress under social distance controls are encouraged to receive psychosocial interventions to improve their mental health.

### Supplementary Information

Supplementary Information.

## Data Availability

All data generated or analyzed in this study are included in this published article and its e-supplemental material.
