# Supplementary material for: The effects of psychosocial and behavioral interventions on depressive and anxiety symptoms during the COVID-19 pandemic: a systematic review and meta-analysis
Source: Sci Rep. 2023 Nov 4;13:19094. doi: 10.1038/s41598-023-45839-0 (PMC10625531; doi:10.1038/s41598-023-45839-0)
Supplement: Supplementary file 1 — Supplementary Information. [file 41598_2023_45839_MOESM1_ESM.docx]

**e-Supplementary Content**

**Supplementary Table 1.** PRISMA checklist.

**Supplementary Table 2.** PubMed search strategy.

**Supplementary Table 3.** Version 2 of the cochrane risk of bias tool (RoB 2) for included RCT studies.

**Supplementary Table 4.** Pooled effect sizes with heterogeneity statistics for psychosocial and behavioral interventions on depressive outcomes.

**Supplementary Table 5.** Pooled effect sizes with heterogeneity statistics of psychosocial and behavioral interventions on anxiety outcomes.

**Supplementary Table 6.** The mean difference of intervention group vs. control group on depressive and anxiety symptoms in each included study.

**Supplementary Table 7.** Univariate meta-regression analyses of moderators of psychosocial and behavioral interventions on depression.

**Supplementary Table 8.** Univariate meta-regression analyses of moderators of psychosocial and behavioral interventions on anxiety.

**Supplementary Table 9.** Multiple meta-regression analyses of the interaction effect between moderators on anxiety.

**Supplementary Figure 1.** Funnel plot of included studies for depression.

**Supplementary Figure 2.** Funnel plot of included studies for anxiety.

**Supplementary Figure 3.** Forest plot of sensitive analysis for depression excluding studies without mentioned length of intervention.

**Supplementary Table 1.** PRISMA checklist.

| **Section and Topic** | **Item #** | **Checklist item** | **Location where item is reported** |
| --- | --- | --- | --- |
| **TITLE** | | |  |
| Title | 1 | Identify the report as a systematic review. | Title page |
| **ABSTRACT** | | |  |
| Abstract | 2 | See the PRISMA 2020 for Abstracts checklist. | Title page |
| **INTRODUCTION** | | |  |
| Rationale | 3 | Describe the rationale for the review in the context of existing knowledge. | P2-3 of manuscript document |
| Objectives | 4 | Provide an explicit statement of the objective(s) or question(s) the review addresses. | P3-4 of manuscript document |
| **METHODS** | | |  |
| Eligibility criteria | 5 | Specify the inclusion and exclusion criteria for the review and how studies were grouped for the syntheses. | P4-5 of manuscript document |
| Information sources | 6 | Specify all databases, registers, websites, organisations, reference lists and other sources searched or consulted to identify studies. Specify the date when each source was last searched or consulted. | P4 of manuscript document |
| Search strategy | 7 | Present the full search strategies for all databases, registers and websites, including any filters and limits used. | e-Supplementary Table 2 |
| Selection process | 8 | Specify the methods used to decide whether a study met the inclusion criteria of the review, including how many reviewers screened each record and each report retrieved, whether they worked independently, and if applicable, details of automation tools used in the process. | P5-6 of manuscript document |
| Data collection process | 9 | Specify the methods used to collect data from reports, including how many reviewers collected data from each report, whether they worked independently, any processes for obtaining or confirming data from study investigators, and if applicable, details of automation tools used in the process. | P6 of manuscript document |
| Data items | 10a | List and define all outcomes for which data were sought. Specify whether all results that were compatible with each outcome domain in each study were sought (e.g. for all measures, time points, analyses), and if not, the methods used to decide which results to collect. | P6 of manuscript document |
|  | 10b | List and define all other variables for which data were sought (e.g. participant and intervention characteristics, funding sources). Describe any assumptions made about any missing or unclear information. | P6 of manuscript document |
| Study risk of bias assessment | 11 | Specify the methods used to assess risk of bias in the included studies, including details of the tool(s) used, how many reviewers assessed each study and whether they worked independently, and if applicable, details of automation tools used in the process. | P6-7 of manuscript document |
| Effect measures | 12 | Specify for each outcome the effect measure(s) (e.g. risk ratio, mean difference) used in the synthesis or presentation of results. | P7 of manuscript document |
| Synthesis methods | 13a | Describe the processes used to decide which studies were eligible for each synthesis (e.g. tabulating the study intervention characteristics and comparing against the planned groups for each synthesis (item #5)). | P7 of manuscript document |
|  | 13b | Describe any methods required to prepare the data for presentation or synthesis, such as handling of missing summary statistics, or data conversions. | P7 of manuscript document |
|  | 13c | Describe any methods used to tabulate or visually display results of individual studies and syntheses. | P7 of manuscript document |
|  | 13d | Describe any methods used to synthesize results and provide a rationale for the choice(s). If meta-analysis was performed, describe the model(s), method(s) to identify the presence and extent of statistical heterogeneity, and software package(s) used. | P7 of manuscript document |
|  | 13e | Describe any methods used to explore possible causes of heterogeneity among study results (e.g. subgroup analysis, meta-regression). | P7 of manuscript document |
|  | 13f | Describe any sensitivity analyses conducted to assess robustness of the synthesized results. | P7 of manuscript document |
| Reporting bias assessment | 14 | Describe any methods used to assess risk of bias due to missing results in a synthesis (arising from reporting biases). | Not appliable |
| Certainty assessment | 15 | Describe any methods used to assess certainty (or confidence) in the body of evidence for an outcome. | P7 of manuscript document |
| **RESULTS** | | |  |
| Study selection | 16a | Describe the results of the search and selection process, from the number of records identified in the search to the number of studies included in the review, ideally using a flow diagram. | P8 & Fig. 1 of manuscript document |
|  | 16b | Cite studies that might appear to meet the inclusion criteria, but which were excluded, and explain why they were excluded. | Fig. 1 of manuscript document |
| Study characteristics | 17 | Cite each included study and present its characteristics. | P8-9 & Table 1 of manuscript document |
| Risk of bias in studies | 18 | Present assessments of risk of bias for each included study. | e-Supplementary Table 3 |
| Results of individual studies | 19 | For all outcomes, present, for each study: (a) summary statistics for each group (where appropriate) and (b) an effect estimates and its precision (e.g. confidence/credible interval), ideally using structured tables or plots. | Fig. 2 & Fig. 3 of manuscript document, and e-Supplementary Table 6 |
| Results of syntheses | 20a | For each synthesis, briefly summarise the characteristics and risk of bias among contributing studies. | P16 of manuscript document |
|  | 20b | Present results of all statistical syntheses conducted. If meta-analysis was done, present for each the summary estimate and its precision (e.g., confidence/credible interval) and measures of statistical heterogeneity. If comparing groups, describe the direction of the effect. | P16-19 & Fig. 2-3 of manuscript document & e-Supplement Table 4 and 5 |
|  | 20c | Present results of all investigations of possible causes of heterogeneity among study results. | P16-20 of manuscript document & e-Supplementary Table 4-8 & Fig. 1-3 |
|  | 20d | Present results of all sensitivity analyses conducted to assess the robustness of the synthesized results. | P20 of manuscript document & e-Supplementary Fig. 3 |
| Reporting biases | 21 | Present assessments of risk of bias due to missing results (arising from reporting biases) for each synthesis assessed. | Not applicable |
| Certainty of evidence | 22 | Present assessments of certainty (or confidence) in the body of evidence for each outcome assessed. | Not applicable |
| **DISCUSSION** | | |  |
| Discussion | 23a | Provide a general interpretation of the results in the context of other evidence. | P20-25 of manuscript document |
|  | 23b | Discuss any limitations of the evidence included in the review. | P26 of manuscript document |
|  | 23c | Discuss any limitations of the review processes used. | P26 of manuscript document |
|  | 23d | Discuss implications of the results for practice, policy, and future research. | P26 of manuscript document |
| **OTHER INFORMATION** | | |  |
| Registration and protocol | 24a | Provide registration information for the review, including register name and registration number, or state that the review was not registered. | P4 of manuscript document |
|  | 24b | Indicate where the review protocol can be accessed, or state that a protocol was not prepared. | P4 of manuscript document |
|  | 24c | Describe and explain any amendments to information provided at registration or in the protocol. | Not applicable |
| Support | 25 | Describe sources of financial or non-financial support for the review, and the role of the funders or sponsors in the review. | P27 of manuscript document |
| Competing interests | 26 | Declare any competing interests of review authors. | P27 of manuscript document |
| Availability of data, code and other materials | 27 | Report which of the following are publicly available and where they can be found: template data collection forms; data extracted from included studies; data used for all analyses; analytic code; any other materials used in the review. | P26 of manuscript document |

*From:*  Page MJ, McKenzie JE, Bossuyt PM, Boutron I, Hoffmann TC, Mulrow CD, et al. The PRISMA 2020 statement: an updated guideline for reporting systematic reviews. BMJ 2021;372:n71. doi: 10.1136/bmj.n71

For more information, visit: <http://www.prisma-statement.org/>

**Supplementary Table 2.** PubMed search strategy.

| Concept | Search | Query |
| --- | --- | --- |
| Psychosocial and behavioral interventions | #1 | "cognitive therapy"[Text Word] OR "behavioral therapy"[Text Word] OR "behavioural therapy"[Text Word] OR "existential therapy"[Text Word] OR "interpersonal therapy"[Text Word] OR "relaxation therapy"[Text Word] OR "narrative therapy"[Text Word] OR "family therapy"[Text Word] OR "relationship therapy"[Text Word] OR "relational therapy"[Text Word] OR "emotion focused"[Text Word] OR "emotionally focused"[Text Word] OR "rational emotive"[Text Word] OR "logotherapy"[Text Word] OR "network therapy"[Text Word] OR "support group"[Text Word] OR "support groups"[Text Word] OR "reality therapy"[Text Word] OR "primal therapy"[Text Word] OR "persuasion therapy"[Text Word] OR "transactional analysis"[Text Word] OR "insight therapy"[Text Word] OR "gestalt therapy"[Text Word] OR "feminist therapy"[Text Word] OR "client centered therapy"[Text Word] OR "sociotherap*"[Text Word] OR "mileau"[Text Word] OR "bibliotherapy"[Text Word] OR (("art therapy"[Text Word] OR "arts therapy"[Text Word]) AND "creative"[Text Word]) OR "music therapy"[Text Word] OR "music listening"[Text Word] OR "autogenic training"[Text Word] OR "autogenic therapy"[Text Word] OR "autogenic relaxation"[Text Word] OR ("eye movement"[Text Word] AND ("desensitization"[Text Word] OR "reprocessing"[Text Word])) OR "biofeedback"[Text Word] OR "guided imagery"[Text Word] OR "stress management"[Text Word] OR "Counseling"[MeSH Terms] OR "meditat*"[Text Word] OR "dream analysis"[Text Word] OR "morita therapy"[Text Word] OR "cotherapy"[Text Word] OR "mirroring"[Text Word] OR "storytelling"[Text Word] OR (("reframing"[Text Word] OR "paradoxical"[Text Word]) AND "technique"[Text Word]) OR "empty chair"[Text Word] OR "role playing"[Text Word] OR "improvisation"[Text Word] OR "active listening"[Text Word] OR ("motivational"[Text Word] AND ("interviewing"[Text Word] OR "therapy"[Text Word])) OR "psychoeducation*"[Text Word] OR "psychodynamic"[Text Word] OR "psychosocial"[Text Word] OR "psychodrama"[Text Word] OR "psychotherap*"[Text Word] OR "Psychotherapy"[MeSH Terms] OR "mind body therapy"[Text Word] OR "Mind-Body Therapies"[Text Word] OR "Mind-Body Therapies"[MeSH Terms] OR "Exercise"[Text Word] OR "Exercise"[MeSH Terms] |
| Depression and anxiety | #2 | "psycholog*"[Text Word] OR "mental"[Text Word] OR "mood"[Text Word] OR "Affect"[MeSH Terms] OR "depress*"[Text Word] OR "Depression"[MeSH Terms] OR "anxiet*"[Text Word] OR "Anxiety"[MeSH Terms] |
| COVID-19 | #3 | "Wuhan coronavirus"[Text Word] OR "Wuhan virus"[Text Word] OR "novel coronavirus"[Text Word] OR "n-CoV"[Text Word] OR "SARS-CoV-2"[Text Word] OR "SARS 2"[Text Word] OR "severe acute respiratory syndrome coronavirus 2"[Text Word] OR "COVID-19"[Text Word] OR "coronavirus disease 2019 virus"[Text Word] OR "2019-nCoV"[Text Word] OR "2019 novel coronavirus"[Text Word] OR "coronavirus*"[Text Word] |
| RCT limitation | #4 | ("clinical"[Title/Abstract] AND "trial"[Title/Abstract]) OR "clinical trials as topic"[MeSH Terms] OR "clinical trial"[Publication Type] OR "random*"[Title/Abstract] OR "random allocation"[MeSH Terms] OR "therapeutic use"[MeSH Subheading] |
| Human limitation | #5 | "animals"[MeSH Terms] NOT "humans"[MeSH Terms] |
| Total | #6 | #1 AND #2 AND #3 AND #4 NOT #5 |

**Supplementary Table 3.** Version 2 of the cochrane risk of bias tool (RoB 2) for included RCT studies.

| **Source** | **Bias arising from the randomization process** | **Bias due to deviations from the intended interventions** | **Bias due to missing outcome data** | **Bias in measurement of the outcome** | **Bias in selection of the reported result** | **Overall**  **bias** |
| --- | --- | --- | --- | --- | --- | --- |
| Beauchamp et al., 2021 ^1^ | Some concerns | Low risk | Low risk | Low risk | Low risk | Some concerns |
| Borrega-Mouquinho et al., 2021 ^2^ | Low risk | Low risk | Some concerns | Low risk | Low risk | Some concerns |
| Chen et al., 2020 ^3^ | Low risk | Low risk | High risk | Low risk | Low risk | High risk |
| Cozzolino et al., 2021 ^4^ | Low risk | Low risk | Some concerns | Low risk | Low risk | Some concerns |
| Cui et al., 2021 ^5^ | Low risk | Low risk | Low risk | Low risk | Low risk | Low risk |
| Egan et al., 2021 ^6^ | Low risk | Low risk | Some concerns | Low risk | Low risk | Some concerns |
| Fan et al., 2021 ^7^ | Some concerns | Low risk | Low risk | Low risk | Low risk | Some concerns |
| Fiol-DeRoque et al., 2021 ^8^ | Some concerns | Low risk | Low risk | Low risk | Low risk | Some concerns |
| Ghazanfarpour et al., 2021 ^9^ | Low risk | Low risk | Some concerns | Low risk | Low risk | Some concerns |
| Gu et al., 2021 ^10^ | Low risk | Low risk | Low risk | Low risk | Low risk | Low risk |
| He et al., 2021 ^11^ | Low risk | Low risk | Low risk | Low risk | Low risk | Low risk |
| Kam et al., 2021 ^12^ | Some concerns | Low risk | Low risk | Low risk | Low risk | Some concerns |
| Kong et al., 2020 ^13^ | Some concerns | Low risk | Low risk | Low risk | Low risk | Some concerns |
| Latino et al., 2021 ^14^ | Some concerns | Low risk | Low risk | Low risk | Low risk | Some concerns |
| Li, 2020 ^15^ | Some concerns | Low risk | Low risk | Low risk | Low risk | Some concerns |
| Li et al., 2020 ^16^ | Low risk | Low risk | Low risk | Low risk | Low risk | Low risk |
| Liang et al., 2021^17^ | Low risk | Low risk | Low risk | Low risk | Low risk | Low risk |
| Liu et al., 2021 ^18^ | Some concerns | Low risk | Low risk | Low risk | Low risk | Some concerns |
| Öner Cengiz et al., 2021 ^19^ | Low risk | Low risk | High risk | Low risk | Low risk | High risk |
| Özlü et al., 2021 ^20^ | Some concerns | Low risk | High risk | Low risk | Low risk | High risk |
| Pan et al., 2021 ^21^ | Low risk | Low risk | Low risk | Low risk | Low risk | Low risk |
| Parizad et al., 2021 ^22^ | Low risk | Low risk | Low risk | Low risk | Low risk | Low risk |
| Puterman et al., 2021 ^23^ | Low risk | Low risk | Low risk | Low risk | Low risk | Low risk |
| Shabahang et al., 2021 ^24^ | Some concerns | Low risk | Low risk | Low risk | Low risk | Some concerns |
| Solianik et al., 2021 ^25^ | Some concerns | Low risk | Low risk | Low risk | Low risk | Some concerns |
| Wahlund et al., 2021 ^26^ | Some concerns | Low risk | Low risk | Low risk | Low risk | Some concerns |
| Wang et al., 2021a ^27^ | Low risk | Low risk | Low risk | Low risk | Low risk | Low risk |
| Wang et al., 2021b ^28^ | Some concerns | Low risk | Low risk | Low risk | Low risk | Some concerns |
| Wilke et al., 2022 ^29^ | Low risk | Low risk | Low risk | Low risk | Low risk | Low risk |
| Yang et al., 2020 ^30^ | Some concerns | Low risk | Low risk | Low risk | Low risk | Some concerns |
| Zhang et al., 2021 ^31^ | Some concerns | Low risk | Low risk | Low risk | Low risk | Some concerns |
| Zhang, 2021 ^32^ | Some concerns | Low risk | Low risk | Low risk | Low risk | Some concerns |
| Zhang & Rao, 2020 ^33^ | Low risk | Low risk | Low risk | Low risk | Low risk | Low risk |
| Zhou et al., 2022 ^34^ | Low risk | Low risk | Low risk | Low risk | Low risk | Low risk |
| Zhu et al., 2020 ^35^ | Low risk | Low risk | Low risk | Low risk | Low risk | Low risk |

**Supplementary Table 4.** Pooled effect sizes with heterogeneity statistics for psychosocial and behavioral interventions on depressive outcomes.

|  | No. of RCTs | Effect size statistics | | | | Heterogeneity statistics | |
| --- | --- | --- | --- | --- | --- | --- | --- |
|  |  | SMD | 95% CI | Median | Range | *I*^2^ | *p-*Value |
| **Participants’ age** | | | | | | |  |
| Non-elderly | 23 | -0.64 | -0.91, -0.38 | -0.44 | -2.57, 0.01 | 89% | <.010 |
| Elderly | 4 | -1.34 | -3.14, 0.47 | -1.80 | -2.41, -0.28 | 95% | <.010 |
| **Clinical conditions of participants** | | | | | | |  |
| Patient with COVID-19 | 10 | -1.02 | -1.66, -0.39 | -0.56 | -2.57, -0.26 | 92% | <.010 |
| Medical profession | 4 | -0.59 | -1.61, 0.43 | -0.48 | -1.51, 0.01 | 92% | <.010 |
| General population | 13 | -0.58 | -0.93, -0.23 | -0.44 | -2.41, -0.08 | 84% | <.010 |
| **Type of intervention** | | | | | | |  |
| Exercise | 7 | -0.62 | -1.21, -0.03 | -0.48 | -2.25, -0.19 | 74% | <.010 |
| CBT | 8 | -0.87 | -1.58, -0.16 | -0.48 | -2.57, -0.26 | 95% | <.010 |
| Psychoeducation | 4 | -0.90 | -2.50, 0.69 | -0.45 | -2.41, -0.46 | 94% | <.010 |
| Mindfulness-based intervention | 5 | -0.62 | -1.29, 0.05 | -0.44 | -1.15, -0.08 | 81% | <.010 |
| Multiple interventions | 3 | -0.46 | -2.00, 1.09 | -0.47 | -1.35, 0.01 | 86% | <.010 |
| **Location** | | | | | | | |
| America | 1 | -0.08 | -0.57, 0.42 | NA | NA | NA | NA |
| Canada | 2 | -0.24 | -0.80, 0.33 | -0.24 | -0.19, 0.28 | 0% | .690 |
| China | 18 | -1.00 | -1.38, -0.61 | -1.02 | -2.57, -0.26 | 90% | <.010 |
| Europe | 4 | -0.24 | -0.59, 0.11 | -0.33 | -0.48, 0.01 | 74% | <.010 |
| West Asia | 1 | -0.40 | -0.81, 0.01 | NA | NA | NA | NA |
| Multiple locations | 1 | -0.33 | -0.63, -0.03 | NA | NA | NA | NA |
| **Delivery mode of the intervention** | | | | | | |  |
| Face-to-face | 14 | -0.97 | -1.47, -0.48 | -0.57 | -2.57, -0.08 | 89% | <.010 |
| Online | 13 | -0.52 | -0.82, -0.22 | -0.40 | -1.89, 0.01 | 90% | <.010 |

**Supplementary Table 5.** Pooled effect sizes with heterogeneity statistics of psychosocial and behavioral interventions on anxiety outcomes.

|  | No. of RCTs | Effect size statistics | | | | Heterogeneity statistics | |
| --- | --- | --- | --- | --- | --- | --- | --- |
|  |  | SMD | 95% CI | Median | Range | *I*^2^ | *p-*Value |
| **Participants’ age** | | | | | | |  |
| Non-elderly | 30 | -0.84 | -1.14, -0.54 | -0.56 | -3.24, 0.23 | 92% | <.010 |
| Elderly | 3 | -1.50 | -3.74, 0.73 | -1.98 | -2.41, -0.28 | 85% | <.010 |
| **Clinical conditions of participants** | | | | | | |  |
| Patient with COVID-19 | 14 | -1.09 | -1.64, -0.54 | -0.89 | -3.24, 0.12 | 93% | <.010 |
| Medical profession | 4 | -0.69 | -1.71, 0.32 | -0.60 | -1.60, -0.08 | 92% | <.010 |
| General population | 15 | -0.79 | -1.21, -0.36 | -0.49 | -2.45, 0.23 | 90% | <.010 |
| **Type of intervention** | | | | | | |  |
| Exercise | 10 | -1.00 | -1.64, -0.36 | -0.74 | -2.45, 0.00 | 90% | <.010 |
| CBT | 10 | -1.05 | -1.77, -0.34 | -0.62 | -3.24, 0.12 | 95% | <.010 |
| Psychoeducation | 4 | -1.08 | -2.20, 0.05 | -0.90 | -2.05, -0.46 | 88% | <.010 |
| Mindfulness-based intervention | 5 | -0.48 | -1.32, 0.37 | -0.40 | -1.60, 0.23 | 88% | <.010 |
| Multiple interventions | 4 | -0.59 | -1.49, 0.30 | -0.64 | -1.49, -0.08 | 86% | <.010 |
| **Location** | | | | | | | |
| America | 1 | 0.23 | -0.27, 0.73 | NA | NA | NA | NA |
| China | 19 | -1.05 | -1.45, -0.65 | -0.90 | -3.24, 0.12 | 92% | <.010 |
| Europe | 6 | -0.58 | -1.29, 0.14 | -0.52 | -2.45, -0.08 | 88% | <.010 |
| West Asia | 5 | -1.16 | -2.24, -0.09 | -0.68 | -2.26, -0.41 | 92% | <.010 |
| Multiple locations | 2 | -0.23 | -3.28, 2.82 | -0.24 | -0.48, 0.00 | 85% | .010 |
| **Delivery mode of the intervention** | | | | | | |  |
| Face-to-face | 18 | -1.02 | -1.47, -0.58 | -0.89 | -3.24, 0.23 | 90% | <.010 |
| Online | 15 | -0.76 | -1.17, -0.35 | -0.51 | -2.45, 0.00 | 94% | <.010 |

**Supplementary Table 6.** The mean difference of intervention group vs. control group on depressive and anxiety symptoms in each included study.

|  | Depressive symptoms | | | Anxiety symptoms | | |
| --- | --- | --- | --- | --- | --- | --- |
| Source | Measurement tool | Mean difference | Effect size | Measurement tool | Mean difference | Effect size |
| Beauchamp et al., 2021 ^1^ | CES-D | -1.86 | -0.39 |  |  |  |
| Borrega-Mouquinho et al., 2021 ^2^ | BDI-13 | -0.11 | -0.04 | STAI | -0.27 | -0.04 |
| Chen et al., 2020 ^3^ | SDS | -10.91 | -2.60 | SAS | -12.00 | -3.00 |
| Cozzolino et al., 2021 ^4^ |  |  |  | STAI | -4.68 | -0.38 |
| Cui et al., 2021 ^5^ | PHQ-9 | -3.13 | -0.79 | GAD-7 | -2.02 | -0.65 |
| Egan et al., 2021 ^6^ | PHQ-9 | -2.47 | -0.46 | GAD-7 | -2.29 | -0.51 |
| Fan et al., 2021 ^7^ | SDS | -3.51 | -0.39 | SAS | 0.94 | 0.10 |
| Fiol-DeRoque et al., 2021 ^8^ | DASS-21 | -0.08 | -0.02 | DASS-21 | -0.63 | -0.22 |
| Ghazanfarpour et al., 2021 ^9^ | HADS | -0.47 | -0.21 | HADS | -0.62 | -0.30 |
| Gu et al., 2021 ^10^ | SDS | -5.51 | -0.63 | SAS | -4.60 | -0.73 |
| He et al., 2021 ^11^ | SCL-90 | -0.32 | -4.91 | SCL-90 | -0.33 | -4.67 |
| Kam et al., 2021 ^12^ | PROMIS | -0.73 | -0.10 | PROMIS | -1.23 | -0.15 |
| Kong et al., 2020 ^13^ | HADS | -3.46 | -1.05 | HADS | -3.77 | -1.03 |
| Latino et al., 2021 ^14^ |  |  |  | QAS-anxiety | -4.07 | -2.47 |
| Li, 2020 ^15^ | SDS | -6.11 | -1.69 | SAS | -7.68 | -1.86 |
| Li et al., 2020 ^16^ | DASS-21 | -0.09 | -0.04 | DASS-21 | -0.90 | -0.24 |
| Liang et al., 2021 ^17^ | PHQ-9 | -3.00 | -0.72 | GAD-7 | -2.77 | -0.78 |
| Liu et al., 2021 ^18^ | HAMA | -7.01 | -1.95 | HAMD | -5.84 | -1.71 |
| Öner Cengiz et al., 2021 ^19^ |  |  |  | BAI | -5.45 | -0.51 |
| Özlü et al., 2021 ^20^ |  |  |  | STAI | -16.62 | -2.44 |
| Pan et al., 2021 ^21^ | SDS | -4.75 | -0.47 | SAS | -5.50 | -0.53 |
| Parizad et al., 2021 ^22^ |  |  |  | STAI | -8.94 | -0.73 |
| Puterman et al., 2021 ^23^ | CES-D | -0.73 | -0.13 |  |  |  |
| Shabahang et al., 2021 ^24^ |  |  |  | CVAQ | -6.64 | -1.80 |
| Solianik et al., 2021 ^25^ | HADS | -1.40 | -0.42 | HADS | -1.60 | -0.68 |
| Wahlund et al., 2021 ^26^ | MADRS-S | -2.59 | -0.36 | GAD-7 | -2.85 | -0.57 |
| Wang et al., 2021a ^27^ |  |  |  | SAS | -10.39 | -0.99 |
| Wang et al., 2021b ^28^ | SDS | -6.62 | -0.96 | SAS | -10.50 | -1.39 |
| Wilke et al., 2022 ^29^ |  |  |  | GAD-7 | 0.30 | -0.06 |
| Yang et al., 2020 ^30^ | DASS-21 | -1.28 | -0.50 | DASS-21 | -1.21 | -0.48 |
| Zhang et al., 2021 ^31^ | SDS | -5.10 | -0.57 | SAS | -3.98 | -0.39 |
| Zhang, 2021 ^32^ | SDS | -5.33 | -1.55 | SAS | -2.24 | -0.95 |
| Zhang & Rao, 2020 ^33^ | SDS | -4.91 | -0.80 | SAS | -8.72 | -1.07 |
| Zhou et al., 2022 ^34^ | PHQ-9 | -3.10 | -0.67 | GAD-7 | -3.40 | -0.74 |
| Zhu et al., 2020 ^35^ | SDS | -15.63 | -3.02 | SAS | -16.39 | -4.09 |

Note: BAI: Beck Anxiety Inventory; BDI: Beck Depression Inventory; CVAQ: COVID-19 Anxiety Questionnaire; CES-D: Center for Epidemiologic Studies Depression Scale; DASS-21: Depression, Anxiety, and Stress Scale-21 Items; GAD-7: General Anxiety Disorder-7; HADS: Hospital Anxiety and Depression Scale; HAMA: Hamilton Rating Scale for Anxiety; HAMD: Hamilton Rating Scale for Depression; MADSR: Montgomery-Asberg Depression Rating Scale; PHQ-9: Patient Health Questionnaire-9; PROMIS: Patient-Reported Outcomes Measurement Information System; QAS: Study Approach Questionnaire (QAS) - Anxiety; SAS: Self-rating Anxiety Scale; SCL-90: Symptom Checklist-90; SDS: Self-rating Depression Scale; SHAI: Short Health Anxiety Inventory; STAI: State-Trait Anxiety Inventory.

**Supplementary Table 7.** Univariate meta-regression analyses of moderators of psychosocial interventions in depression.

| **Moderators**  **(k=No. of studies)** | **Coefficient**  **(SE)** | **95%CI** | | **τ^2^** | **R^2^ (%)** | ***I*^2^ (%)** | ***p*-Value** |
| --- | --- | --- | --- | --- | --- | --- | --- |
|  |  | **Lower** | **Upper** |  |  |  |  |
| **Continuous** |  | | | | | | |
| Participant’s age, years (k=24) | 0.01 (0.01) | -0.03 | 0.01 | 0.358 | 0.00 | 91.54 | .298 |
| Participant sex, % of female (k=25) | 0.01 (0.01) | 0.00 | 0.02 | 0.286 | 13.03 | 89.44 | .191 |
| Sessions of intervention, times (k=14) | 0.00 (0.01) | 0.01 | 0.01 | 0.000 | 0.00 | 9.79 | .741 |
| **Categorical** |  | | | | | | |
| Location |  | | | | | | |
| China (k=18) | Referent | | | | | | |
| America (k=1) | 0.91 (0.68) | -0.46 | 2.34 | 0.305 | 7.15 | 88.47 | .194 |
| Canada (k=2) | 0.75 (0.49) | -0.27 | 1.77 | 0.305 | 7.15 | 88.47 | .142 |
| Europe (k=4) | 0.72 (0.36) | -0.03 | 1.47 | 0.305 | 7.15 | 88.47 | .058 |
| West Asia (k=1) | 0.59 (0.67) | -0.79 | 1.97 | 0.305 | 7.15 | 88.47 | .386 |
| Multiple locations (k=1) | 0.66 (0.65) | -0.68 | 2.01 | 0.305 | 7.15 | 88.47 | .319 |
| Type of control group |  |  |  |  |  |  |  |
| Active (k=9) | Referent | | | | | | |
| Non-active (k=18) | -0.11 (0.30) | -0.72 | 0.50 | 0.345 | 0.00 | 90.33 | .714 |
| Severity of baseline depression |  | | | | | | |
| Mild to moderate (k=21) | Referent | | | | | | |
| Severe (k=6) | -0.59 (0.33) | -1.27 | 0.09 | 0.324 | 1.37 | 90.77 | .087 |

**Supplementary Table 8.** Univariate meta-regression analyses of moderators of psychosocial interventions in anxiety.

| **Moderators**  **(k=No. of studies)** | **Coefficient**  **(SE)** | **95%CI** | | **τ^2^** | **R^2^ (%)** | ***I*^2^ (%)** | ***p*-Value** |
| --- | --- | --- | --- | --- | --- | --- | --- |
|  |  | Lower | Upper |  |  |  |  |
| **Continuous** |  | | | | | | |
| Participant’s age, years (k=31) | 0.00(0.01) | -0.03 | 0.02 | 0.453 | 0.00 | 92.85 | .890 |
| Participant sex, % of female (k=31) | 0.01 (0.01) | 0.00 | 0.03 | 0.379 | 11.94 | 91.62 | .067 |
| Session of intervention, times (k=18) | 0.01 (0.02) | -0.03 | 0.04 | 0.510 | 0.00 | 92.95 | .663 |
| **Categorical** |  | | | | | | |
| Location |  | | | | | | |
| China (k=19) | Referent | | | | | | |
| America (k=1) | 1.28 (0.83) | -0.41 | 2.97 | 0.399 | 5.15 | 91.08 | .132 |
| Europe (k=6) | 0.39 (0.38) | -0.39 | 1.16 | 0.399 | 5.15 | 91.08 | .314 |
| West Asia (k=5) | -0.11 (0.40) | -0.94 | 0.71 | 0.399 | 5.15 | 91.08 | .780 |
| Multiple locations (k=2) | 0.81 (0.17) | -0.37 | 2.00 | 0.399 | 5.15 | 91.08 | .171 |
| Type of control group |  | | | | | | |
| Active (k=12) | Referent | | | | | | |
| Non-active (k=21) | -0.28 (0.30) | -0.88 | 0.35 | 0.419 | 0.31 | 91.91 | .380 |
| Severity of baseline depression |  | | | | | | |
| Mild to moderate (k=17) | Referent | | | | | | |
| Severe (k = 16) | -0.24 (0.33) | -0.92 | 0.44 | 0.440 | 0.00 | 92.33 | .668 |

**Supplementary Table 9.** Multiple meta-regression analyses of the interaction effect between moderators on anxiety.

|  | **Coefficient**  **(SE)** | **95% CI** | | ***p*-Value** |
| --- | --- | --- | --- | --- |
|  |  | **Lower** | **Upper** |  |
| **Correlation** |  |  |  |  |
| **Model 1. Intervention and gender** |  |  |  |  |
| Intercept | -0.02 (0.02) | -0.05 | 0.02 | .337 |
| (CBT vs. Mindfulness) ×Gender | 0.05 (0.02) | 0.00 | 0.09 | .034 |
| (Exercise vs. Mindfulness) ×Gender | 0.05 (0.02) | 0.00 | 0.10 | .032 |
| (Multiple vs. Mindfulness) ×Gender | 0.03 (0.03) | -0.03 | 0.09 | .316 |
| (Psychoeducation vs. Mindfulness) ×Gender | -0.01 (0.03) | -0.07 | 0.06 | .818 |
| **Model 2. Clinical conditions and gender** |  |  |  |  |
| Intercept | 0.03 (0.01) | 0.00 | 0.06 | .025 |
| (Medical vs. General) ×Gender | -0.03 (0.02) | -0.07 | 0.00 | .055 |
| (Patient vs. General) ×Gender | 0.02 (0.03) | -0.04 | 0.08 | .558 |
| **Heterogeneity** | **τ^2^** | **R^2^ (%)** | | ***I*^2^ (%)** |
| Model 1 | 0.531 | 15.94 | | 92.90 |
| Model 2 | 0.544 | 13.91 | | 93.55 |

Note: The second moderator in each comparison model is the referent (e.g., CBT vs. Mindfulness, Mindfulness is the referent), and the gender means the proportion of female participants.

**
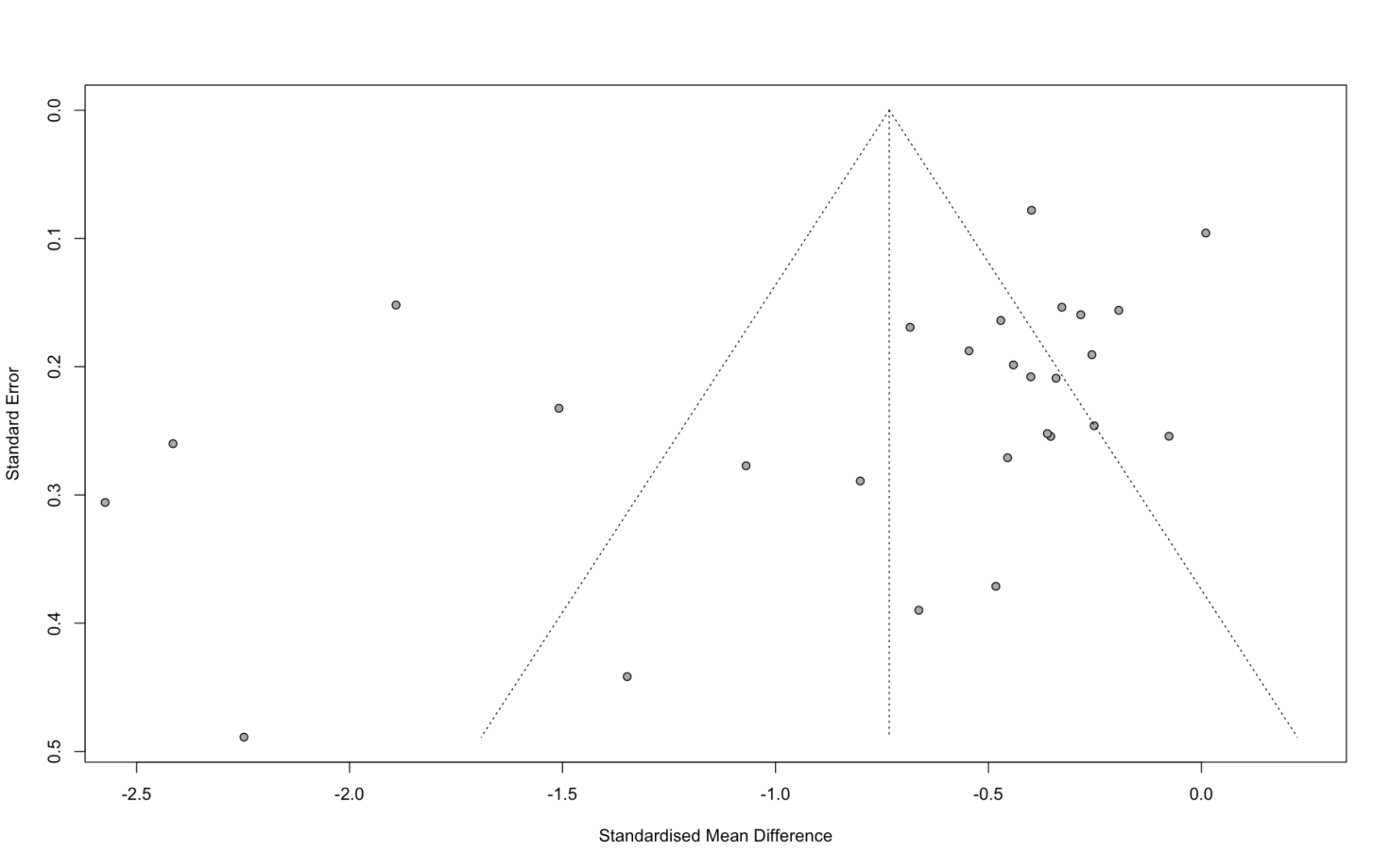
**

**Supplementary Fig. 1.** Funnel plot of included studies for depression.

**
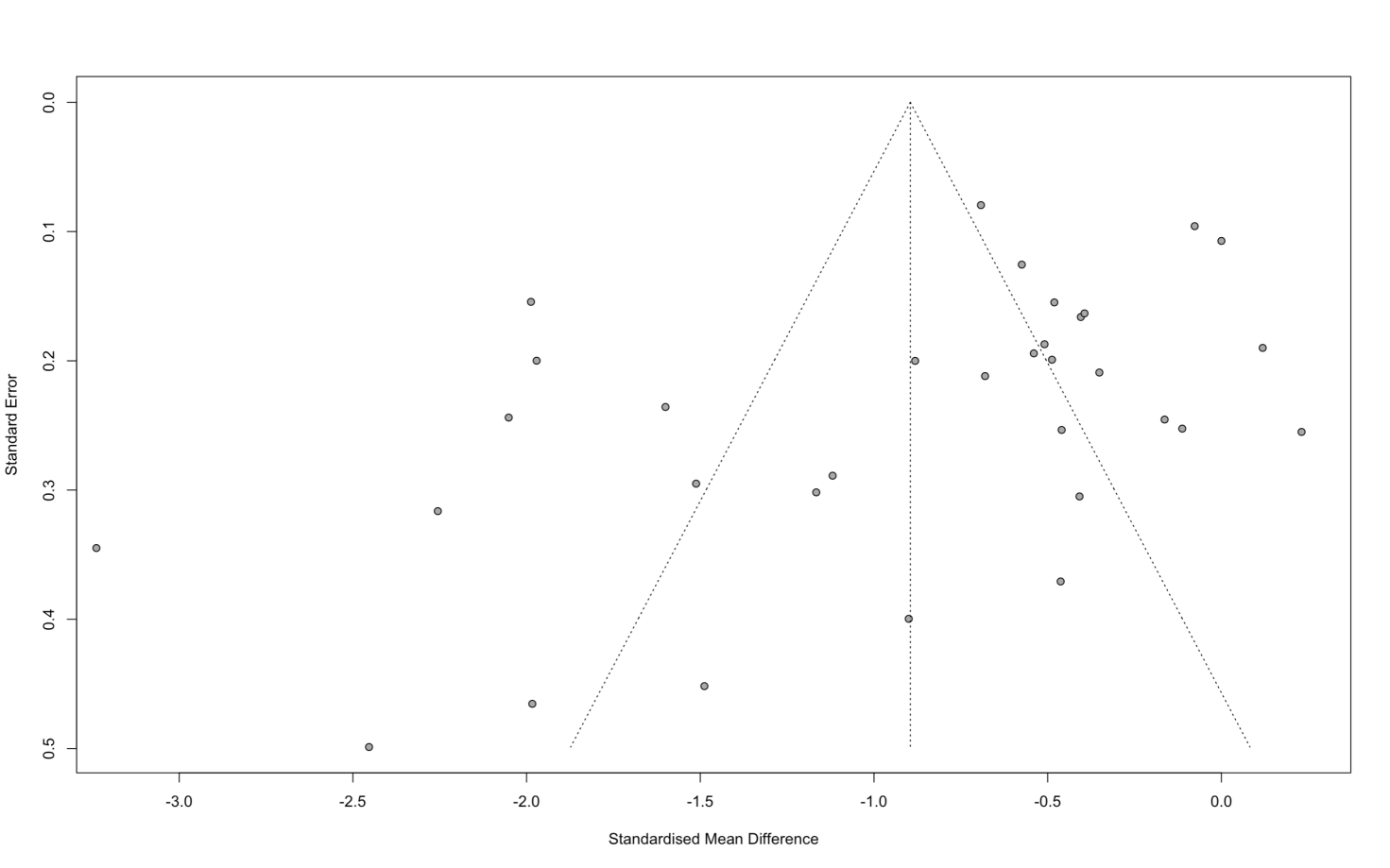
**

**Supplementary Fig. 2.** Funnel plot of included studies for anxiety.


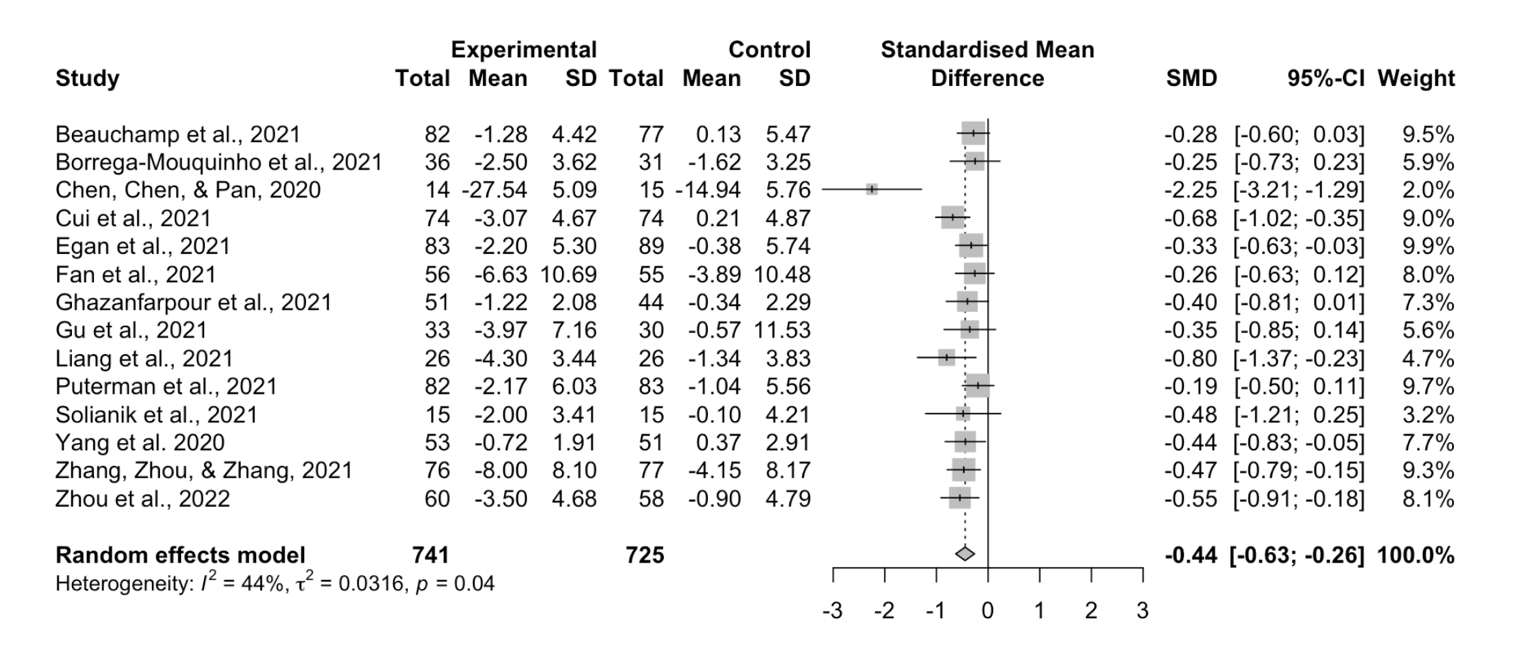


**Supplementary Fig. 3.** Forest plot of sensitive analysis for depression excluding studies without mentioned length of intervention.

**References**

1. Beauchamp, M. R. *et al.* Online-Delivered Group and Personal Exercise Programs to Support Low Active Older Adults' Mental Health During the COVID-19 Pandemic: Randomized Controlled Trial. *J Med Internet Res* **23**, e30709, doi:10.2196/30709. (2021).

2. Borrega-Mouquinho, Y., Sánchez-Gómez, J., Fuentes-García, J. P., Collado-Mateo, D. & Villafaina, S. Effects of High-Intensity Interval Training and Moderate-Intensity Training on Stress, Depression, Anxiety, and Resilience in Healthy Adults During Coronavirus Disease 2019 Confinement: A Randomized Controlled Trial. *Front Psychol* **12**, 643069, doi:10.3389/fpsyg.2021.643069. (2021).

3. Chen, X. Y., Chen, Y. P. & Pan, X. J. The effect of baduanjin on bed on the mental health of patients with novel coronavirus pneumonia (COVID-19). *Chin J Convalescent Med* **29**, 1137-1139, doi:10.13517/j.cnki.ccm.2020.11.006. (2020).

4. Cozzolino, M., Celia, G., Girelli, L. & Limone, P. Effects of the Brain Wave Modulation Technique Administered Online on Stress, Anxiety, Global Distress, and Affect During the First Wave of the COVID-19 Pandemic: A Randomized Clinical Trial. *Front Psychol* **12**, doi:10.3389/fpsyg.2021.635877. (2021).

5. Cui, X. M., Xu, D. D., Miao, Y. D., Liu, M. Z. & Cheng, Q. M. Effect of online mindful stress reduction therapy on relieving the stress of the masses during outbreak of COVID-19. *Chin J Health Psychol* **29**, 259-262, doi:10.13342/j.cnki.cjhp.2021.02.021. (2021).

6. Egan, S. J. *et al.* Unguided low intensity cognitive behaviour therapy for anxiety and depression during the COVID-19 pandemic: A randomised trial. *Behav Res Ther* **144**, 103902, doi:10.1016/j.brat.2021.103902 (2021).

7. Fan, Y. *et al.* The effects of narrative exposure therapy on COVID-19 patients with post-traumatic stress symptoms: A randomized controlled trial. *J Affect Disord* **293**, 141-147, doi:10.1016/j.jad.2021.06.019. (2021).

8. Fiol-DeRoque, M. A. *et al.* A Mobile Phone-Based Intervention to Reduce Mental Health Problems in Health Care Workers During the COVID-19 Pandemic (PsyCovidApp): Randomized Controlled Trial. *JMIR Mhealth Uhealth* **9**, e27039, doi:10.2196/27039. (2021).

9. Ghazanfarpour, M. *et al.* Investigating the effectiveness of tele-counseling for the mental health of staff in hospitals and COVID-19 clinics: a clinical control trial. *Trends Psychiatry Psychother*, doi:10.47626/2237-6089-2020-0176. (2021).

10. Gu, Y. Q., Li, C. F., Song, Y., Lu, G. L. & Y, J. Effects of mindfulness-based stress reduction using Tencent Meeting on anxiety and depression of isolated patients with COVID-19. *Chinese Nurs Manage* **21**, 707-712, doi:10.3969/j.issn.1672-1756.2021.05.015. (2021).

11. He, P. *et al.* The impact of the integrated psychotherapy intervention model on the mental health of the elderly in a nursing homes during the novel coronavirus pneumonia epidemic. *Psycholo Monthly* **16**, 3-4, doi:10.19738/j.cnki.psy.2021.05.002. (2021).

12. Kam, J. W. Y. *et al.* Daily mindfulness training reduces negative impact of COVID-19 news exposure on affective well-being. *Psychol Res*, 1-12, doi:10.1007/s00426-021-01550-1. (2021).

13. Kong, X. *et al.* Effect of Psychological–Behavioral Intervention on the Depression and Anxiety of COVID-19 Patients. *Front Psychiatry* **11**, doi:10.3389/fpsyt.2020.586355 (2020).

14. Latino, F., Fischetti, F., Cataldi, S., Monacis, D. & Colella, D. The impact of an 8-weeks at-home physical activity plan on academic achievement at the time of covid-19 lock-down in italian school. *Sustainability (Switzerland)* **13**, doi:10.3390/su13115812. (2021).

15. Li, H. The application of positive stress reduction in frontline nurses in the prevention and control of novel coronavirus pneumonia outbreak. *Qinghai Med J* **50**, 40-42 (2020).

16. Li, J. Z. *et al.* The Effect of Cognitive Behavioral Therapy on Depression, Anxiety, and Stress in Patients With COVID-19: A Randomized Controlled Trial. *Front Psychiatry* **11**, doi:10.3389/fpsyt.2020.580827. (2020).

17. Liang, L. *et al.* Effect of dialectical behavior group therapy on the anxiety and depression of medical students under the normalization of epidemic prevention and control for the COVID-19 epidemic: a randomized study. *Ann Palliat Med* **10**, 10591-10599, doi:10.21037/apm-21-2466. (2021).

18. Liu, Z. F. *et al.* The Efficacy of Computerized Cognitive Behavioral Therapy for Depressive and Anxiety Symptoms in Patients With COVID-19: Randomized Controlled Trial. *J Med Internet Res* **23**, doi:10.2196/26883. (2021).

19. Öner Cengiz, H., Ayhan, M. & Güner, R. Effect of deep breathing exercise with Triflo on dyspnoea, anxiety and quality of life in patients receiving covid‐19 treatment: A randomized controlled trial. *J Clin Nurs*, doi:10.1111/jocn.16171. (2021).

20. Özlü, İ., Öztürk, Z., Karaman Özlü, Z., Tekin, E. & Gür, A. The effects of progressive muscle relaxation exercises on the anxiety and sleep quality of patients with COVID-19: A randomized controlled study. *Perspect Psychiatr Care*, doi:10.1111/ppc.12750. (2021).

21. Pan, Y. C. *et al.* Effects of psychological nursing on improvement of anxiety and depression in 64 patients with suspected COVID-19. *Chin J Otorhinolaryngol Integr Med* **29**, 141-144, doi:10.16542/j.cnki.issn.1007-4856.2021.02.014. (2021).

22. Parizad, N. *et al.* Effect of guided imagery on anxiety, muscle pain, and vital signs in patients with COVID-19: A randomized controlled trial. *Complement Ther Clin Pract* **43**, 101335, doi:10.1016/j.ctcp.2021.101335. (2021).

23. Puterman, E. *et al.* COVID-19 Pandemic and Exercise (COPE) trial: a multigroup pragmatic randomised controlled trial examining effects of app-based at-home exercise programs on depressive symptoms. *Br J Sports Med*, doi:10.1136/bjsports-2021-104379. (2021).

24. Shabahang, R., Aruguete, M. S. & McCutcheon, L. Video-based cognitive-behavioral intervention for COVID-19 anxiety: a randomized controlled trial. *Trends Psychiatry Psychother* **43**, 141-150, doi:10.47626/2237-6089-2020-0056. (2021).

25. Solianik, R., Mickevičienė, D., Žlibinaitė, L. & Čekanauskaitė, A. Tai chi improves psychoemotional state, cognition, and motor learning in older adults during the COVID-19 pandemic. *Exp Gerontol* **150**, 111363, doi:10.1016/j.exger.2021.111363. (2021).

26. Wahlund, T. *et al.* Brief Online Cognitive Behavioural Intervention for Dysfunctional Worry Related to the COVID-19 Pandemic: A Randomised Controlled Trial. *Psychother Psychosom* **90**, 191-199, doi:10.1159/000512843. (2021).

27. Wang, H., Lan, H. L., Peng, J. Y. & Zhou, L. The Application of Mental Intervention in Patients Left in Fever Clinic for Medical Observation in The Novel Coronavirus Pneumonia Epidemic Period. *Modern Nurs* **28**, 154-156, doi:10.19793/j.cnki.1006-6411.2021.06.060. (2021).

28. Wang, X. L., Zhang, N. J., Zhang, M. & Qiao, Y. Observation on the effectiveness of psychological care for 56 patients with novel coronavirus pneumonia. *Contemp Med* **27**, 179-180, doi:10.3969/j.issn.1009-4393.2021.25.079. (2021).

29. Wilke, J. *et al.* Train at home, but not alone: a randomised controlled multicentre trial assessing the effects of live-streamed tele-exercise during COVID-19-related lockdowns. *Br J Sports Med*, doi:10.1136/bjsports-2021-104994. (2022).

30. Yang, L. *et al.* Effect of mindfulness-based stress reduction on college students mental state and sleep during the epidemic of COVID-19. *Chin J Health Psychol* **28**, 1813-1817, doi:10.13342/j.cnki.cjhp.2020.12.013. (2020).

31. Zhang, J., Zhou, Z. & Zhang, W. Intervention Effect of Research-based Psychological Counseling on Adolescents' Mental Health during the COVID-19 Epidemic. *Psychiatr Danub* **33**, 209-216, doi:10.24869/psyd.2021.209. (2021).

32. Zhang, L. The effect of traditional fitness gongfu on the psychological state of college students during the new crown epidemic. *J Yanan Univ (Natural Science Edition)* **40**, 94-98, doi:10.13876/J.cnki.ydnse.2021.01.094. (2021).

33. Zhang, Y. H. & Rao, J. F. Effect of fitness qigong Yi Jin Jing on the psychological status of patients with novel coronavirus pneumonia. *Pract Clin Med* **21**, 81-83, doi:10.13764/j.cnki.lcsy.2020.08.027. (2020).

34. Zhou, K. *et al.* Positive impacts of e-aid cognitive behavioural therapy on the sleep quality and mood of nurses on site during the COVID-19 pandemic. *Sleep Breath*, 1-5, doi:10.1007/s11325-021-02547-1. (2022).

35. Zhu, L. H., Huang, M., Li, M. & Wang, M. Application of cognitive behavioral intervention in psychological intervention of COVID-19 patients. *Infectious Disease Inf* **33**, 452-455, doi:10.3969/j.issn.1007-8134.2020.05.017. (2020).
